# Deciphering the microbiological mechanism of Tongxie Yaofang in treating IBS-D: a multimodal mechanistic study in mice integrating network pharmacology, computational simulation, and 16S rRNA sequencing

**DOI:** 10.3389/ebm.2025.10725

**Published:** 2025-10-03

**Authors:** Donglin Yu, Qianghong Tian, Junxi Shen, Leyao Fang, Zhoujin Tan, Ying Cai

**Affiliations:** School of Traditional Chinese Medicine, Hunan University of Chinese Medicine, Changsha, China

**Keywords:** Tongxie Yaofang, network pharmacology, molecular dynamics, IBS-D, intestinal microbiota

## Abstract

Irritable bowel syndrome with diarrhea (IBS-D), associated with the traditional Chinese medicine (TCM) pattern of liver hyperactivity with spleen deficiency pattern, lacks effective Western treatments. The modern biological relevance of the “intestine–liver–bile acid” axis aligns with this TCM concept, and interactions between intestinal microbiota and diarrhea remain unclear. Network pharmacology, molecular docking, and molecular dynamics were applied to elucidate the mechanisms and compound–target stability of Tongxie Yaofang. An IBS-D mouse model was established using *Senna alexandrina* Mill. combined with confinement stress. Histopathological changes in the liver and spleen were assessed by hematoxylin–eosin (HE) staining, and enzyme-linked immunosorbent assay (ELISA) was performed to quantify total bile acid levels in serum and liver. Ultimately, 16S rRNA high-throughput sequencing was employed to identify predominant and distinctive bacterial species. Network pharmacology and molecular docking revealed that Tongxie Yaofang acts primarily through the TNF-α and IL-17 pathways. Molecular dynamics confirmed strong binding affinities between active compounds (naringenin, divaricatol, and kaempferol) and target proteins. *In vivo*, Tongxie Yaofang alleviated colonic inflammation, increased serum bile acid levels, reduced hepatic bile acid concentrations, and increased intestinal microbial diversity and abundance. The therapeutic effects of Tongxie Yaofang on IBS-D are mechanistically linked to its multi-target actions, including suppression of inflammatory responses, inhibition of pathogenic bacterial overgrowth, restoration of immune homeostasis, and modulation of intestinal microbiota composition toward a probiotic-enriched community.

## Graphical Abstract

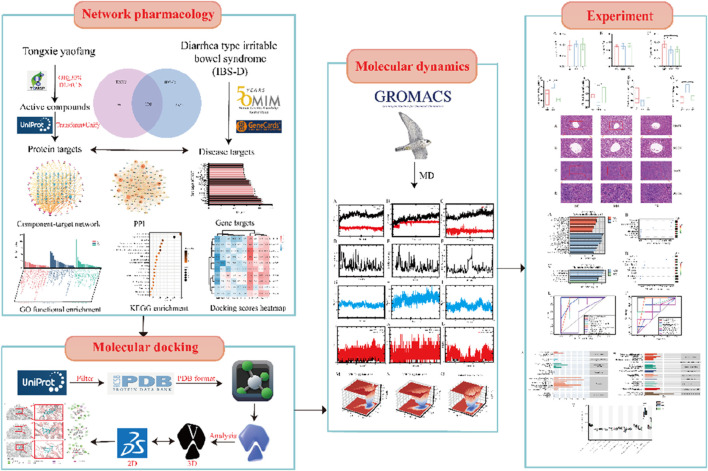

## Impact statement

The active components of Tongxie Yaofang and the effective target of IBS-D were screened, and the consistency of “disease-syndrome-prescription” was confirmed by computer simulation (molecular dynamics). At the same time, the study of microbiology shows the intestinal microecological mechanism of IBS-D occurrence and treatment, which provides theoretical and experimental basis for interpreting the axis of “intestine-liver-bile acid” from the perspective of bile acid, and draws lessons from the clinical thinking of “treating intestine with liver disease” and “treating liver with intestinal disease.”

## Introduction

Irritable bowel syndrome (IBS) is a gastrointestinal condition characterized by abdominal discomfort, bloating, irregular bowel movements, and atypical stool consistency. Epidemiological studies indicate an increasing global prevalence, impacting approximately 5% of Asians and 10%–15% of Europeans and South Americans [1, 2]. Among the four acknowledged IBS subtypes, diarrhea-predominant irritable bowel syndrome (IBS-D) is the most prevalent, representing 31.5% of patients [3]. Research indicates that imbalances in the intestinal microbiota and disturbances in bile acid metabolism are key biological factors contributing to IBS-D, although the precise mechanisms remain unclear [4]. Current treatment strategies primarily include lifestyle modification, psychotherapy, and pharmacotherapy aimed at symptom relief. However, frequently used medications—such as antispasmodics, antidiarrheals and bile acid sequestrants—are limited by side effects, high costs, and a high rate of relapse [5]. Notably, most Western medicines focus on symptom suppression and fail to address the underlying pathophysiology of IBS-D.

Traditional Chinese medicine (TCM), grounded in the principles of holistic regulation and pattern differentiation, offers multitarget and multipathway interventions aimed at restoring systemic balance. Tongxie Yaofang, a classical TCM formula consisting of *Atractylodes macrocephala*, *Saposhnikovia divaricata*, Paeoniae Radix Alba, and Citri Reticulatae Pericarpium, is widely used to treat diarrhea associated with liver hyperactivity with spleen deficiency pattern. Modern pharmacological studies have shown that Tongxie Yaofang has liver-soothing, spleen-strengthening, dampness-dispelling, and antidiarrheal effects [6]. It is commonly used for treating emotionally related gastrointestinal disorders such as ulcerative colitis, IBS, and gastric ulcers and has anti-inflammatory, immunoregulatory, and antitumour properties. However, there is a lack of systematic investigations into its mechanisms of action in IBS-D, particularly concerning its molecular targets, metabolic pathways, and effects on the intestinal microbiota.

Network pharmacology is a systems-level approach that integrates pharmacological data to predict the active ingredients, potential targets, and related pathways of complex herbal formulations. It enables the construction of drug–target–disease interaction networks and facilitates functional pathway analysis [7]. Molecular docking simulates the binding of ligands to target proteins, predicting the interaction modes and affinities of drug–target pairs [8]. Molecular dynamics further refines this analysis by simulating receptor–ligand movements via Newtonian mechanics, thereby assessing binding stability over time [9]. These methods, which emphasize multicomponent and multitarget synergy, are particularly suitable for elucidating the pharmacological mechanisms of complex formulae such as Tongxie Yaofang.

The intestinal microbiota, which is considered a “potential organ,” is integral to health and disease [10]. Diarrhea is frequently associated with intestinal dysbiosis and compromised mucosal barrier function, enabling the transfer of microbial constituents and metabolites into systemic circulation. The intestinal microbiota plays a crucial role in the operation of the “intestine–liver–bile acid” axis, serving as a mediator of host–microbe interactions in both healthy and pathological conditions [11]. Bile acids, which function as signaling molecules, engage in the intestine-liver communication and may contribute to the onset of diarrhea, which is linked to liver hyperactivity with spleen deficiency pattern.

This study initially utilized network pharmacology to elucidate the molecular pathways by which Tongxie Yaofang may confer therapeutic benefits for IBS-D. Molecular docking and molecular dynamics simulations were performed to assess the binding affinities between active drugs and disease-associated protein targets, establishing a theoretical basis for clinical application. *In vivo* investigations utilizing an IBS-D mice model were conducted to evaluate alterations in corticotropin-releasing hormone (CRH), motilin (MTL), and total bile acid (TBA) concentrations through enzyme-linked immunosorbent assay (ELISA). Histopathological alterations in the colonic tissue were assessed using hematoxylin and eosin staining. Additionally, high-throughput sequencing of the 16S rRNA gene was conducted to assess the impact of Tongxie Yaofang on the variety and abundance of the colonic mucosal microbiota. Prominent and distinctive bacterial taxa were identified to investigate the microbiological mechanisms underlying their effectiveness through the intestine–liver–bile acid axis.

## Materials and methods

### Network pharmacology analysis

#### Screening of candidate compounds in Tongxie Yaofang

Utilzing the Traditional Chinese Medicine Systems Pharmacology Database and Analysis Platform (TCMSP^1^), we identified active compounds from the four constituent herbs of Tongxie Yaofang—*A. macrocephala* Koidz., *S. divaricata* Schischk., Paeoniae Radix Alba, Citri Reticulatae Pericarpium. The selection criteria established were oral bioavailability (OB) ≥ 30% and drug-likeness (DL) ≥ 0.18.

#### Intersection of Tongxie Yaofang targets and IBS-D-related targets

The potential compounds’ targets were sourced from TCMSP and the Search Tool for the Retrieval of Interaction Gene/Proteins (STRING)^2^. The results were correlated with the Universal Protein (UniProt)^3^ to provide official protein target information. Therapeutic targets for IBS-D were identified by querying the keywords “IBS-D” and “diarrhea irritable bowel syndrome” in the Human Gene Compendium (GeneCards)^4^ and the Online Mendelian Inheritance in Man (OMIM)^5^.

#### GO enrichment and KEGG pathway analyses

Gene Ontology (GO) and Kyoto Encyclopedia of Genes and Genomes (KEGG) pathway enrichment analyses were performed via DAVID version 2024Q4^6^. Enrichment was deemed statistically significant at *p* < 0.05 and FDR <0.05. The outcomes were illustrated using a bioinformatics platform^7^.

#### Network construction and analysis

To thoroughly elucidate the molecular mechanisms of Tongxie Yaofang in the treatment of IBS-D, a “compound‒target” network and a protein‒protein interaction (PPI) network were developed via Cytoscape software (version 3.9.1). The Network Analyzer plugin was utilized to analyze topological properties.

### Molecular docking

Molecular docking was utilized to confirm essential targets identified through network pharmacology and to predict the binding locations and affinities between ligands and protein targets [12, 13]. Small-molecule ligands were acquired from the TCMSP or PubChem databases^8^ in MOL2 format. Protein crystal structures were acquired from the RCSB Protein Data Bank^9^ in PDB format [14]. Water molecules and existing ligands were eliminated via PyMOL, and the proteins were prepared with AutoDock Tools 1.5.7, which included hydrogenation and conversion to PDBQT format. Ligands were also hydrogenated and ionized prior to docking.

Docking was performed via AutoDock Vina, which generates 20 conformations per ligand‒target pair [15]. The best binding conformation (lowest binding energy) was selected for further analysis. A heatmap of binding affinities was generated via Python 3.13.

### Molecular dynamics simulation

Molecular dynamics (MD) simulations were conducted to evaluate the dynamic interactions and binding stability between proteins and ligands. Based on the docking results, three foremost ligand–protein pairs were identified: protein ID:5KIR with naringenin (MOL004328), protein ID:5KIR with divaricatol (MOL011740), and protein ID:8JOW with kaempferol (MOL000422). In this study, GROMACS 2022.5 was used for molecular dynamics simulation [12, 16, 17]. The ambient pH was established at 7.35, the CHARMM36 force field was employed for protein topology, and the ligand topology was produced via the CGenFF service. The system was solvated with TIP3P water, and ions (Na^+^, H^+^) were added to neutralize the system. The simulation steps are as follows:

(1) Energy minimization: First, proteins were constrained, and energy minimization of ligands was performed via the steepest descent algorithm (step size = 0.01, 5000 cycles).
(2) The balance of the system: Create an index file and build the normalization visualization tool (NVT) ensemble: 100 ps at 300 K for isothermal, isochoric equilibrium. The system was then set to isothermal and isobaric equilibrium within 100 ps in the nuclear nonproliferation treaty (NPT) ensemble parameters, with a time step of 2 fs, and the coordinates were saved every 1.0 ps.
(3) Dynamic simulation: The system performs a molecular dynamics simulation for 100 ns at a constant temperature (300 K) and constant pressure (1 bar) with a time step of 2 fs, and the coordinates are saved every 10.0 ps.

The simulation stability was evaluated by the root mean square deviation (RMSD), the radius of gyration (Rg), the root mean square fluctuation (RMSF), and hydrogen bond analyses. Gibbs free energy landscapes for each complex were calculated and visualized via the DuIvyTools Python library [18].

### Drugs and treatments

*Senna alexandrina* Mill. (Guangxi, batch no.2311300022) was used to induce diarrhea. The composition of Tongxie Yaofang included Citri Reticulatae Pericarpium (Hunan, batch no. 240701), *Saposhnikoviae divaricata* (Hebei, batch no.240502), Paeoniae Radix Alba (Anhui, batch no.SN24082701), and *Atractylodis Macrocephalae* (Zhejiang, batch no.SX24092302). All decoction pieces were purchased from Jiuzhitang Pharmacy, Changsha, Hunan Province, China.

A total of 100 g of *Senna alexandrina* Mill. were immersed in boiling water for 20 min, filtered through sterile gauze, and the filtrate was collected as the first extract. The residue was subjected to a second extraction under the same conditions, and the filtrates were combined. The combined solution was concentrated at 80 °C using a rotary evaporator to obtain an aqueous extract at 100% concentration (1 g/mL), which was stored at 4 °C until use.

Herbs including stir-fried *Atractylodis Macrocephalae* (8 g), Paeoniae Radix Alba (12 g), Citri Reticulatae Pericarpium (9 g), and *Saposhnikoviae Radix* (6 g) were soaked in water for 15–20 min. The mixture was boiled over high heat and simmered for 30 min, twice in succession. The two extracts were filtered through sterile gauze, combined, and concentrated to a final crude drug concentration of 0.25 g/mL. The decoction was stored at 4 °C until use [7].

### Reagents

Mouse total bile acid ELISA kit (batch no. JM-11614M2, Jiangsu Jingmei Biological Technology Co., Ltd., China); mouse corticotropin-releasing hormone ELISA kit (batch no. JM-02792M2, Jiangsu Jingmei Biological Technology Co., Ltd., China); mouse motilin ELISA kit (batch no. JM-02775M2, Jiangsu Jingmei Biological Technology Co., Ltd., China).

### Animals and feed

Thirty SPF-grade, 4-week-old female Kunming mice, each weighing 20 ± 2 g, were acquired from Hunan Slake Jingda Experimental Animal Co., Ltd. [19]. All animals were maintained at the Experimental Animal Center of Hunan University of Chinese Medicine [SCXK (Xiang) 2019--0009] under controlled conditions: temperature 23–25 °C, relative humidity 47–53%, and a standard 12-h light/dark cycle (light from 07:00 to 19:00; dark from 19:00 to 07:00). The Experimental Animal Ethics Committee of Hunan University of Chinese Medicine examined and approved the experimental protocol [Ethics Approval Number: HNUCM21-2409-14].

The mice were fed a clean, pollutant-free standard diet manufactured by Beijing Huafukang Biotechnology Co., Ltd. and supplied by the Experimental Animal Center. The feed certification number is (2024) 06076. The detailed composition and proportions of the diet are shown in Table 1.

**TABLE 1:** Composition and proportions of mouse breeding feed.

Ingredients	Content	Ingredients	Content
Crude Protein	≥200 g	Calcium	10–18 g
Crude fat	≥40 g	Phosphorus	6–12 g
Moisture	≤100 g	Lysine	≥13.2 g
Coarse ash powder	≤80 g	Methionine + Cystine	≥7.8 g
Crude Fiber	≤50 g	Vitamin E	≥120IU

### Experimental design

Modeling phase: Following 5 days of adaptive feeding, 30 mice were randomly allocated into two groups: a normal control (MC) group (n = 10) and a model (MM) group (n = 20). The modeling procedure was adapted on the basis of a previously published method [20]. All the mice were fasted for 12 h before each gavage. Each morning at 08:30, the mice in the MM group were orally supplied 0.35 mL of *Senna alexandrina* Mill. to produce spleen deficiency diarrhea, while the MC group received an equivalent dose of distilled water. At 15:00, the mice were subjected to restraint stress by placement in centrifuge tubes to restrict limb movement, while tail-clamping stimulation with long hemostatic clips was applied for 1 h. The procedure involved 15-min intermittent rest periods and was repeated four times. During the modeling phase, mice in both the MM and TX groups were subjected to fasting and water deprivation for seven consecutive days, whereas mice in the MC group had free access to food and water.

**FIGURE 1:**
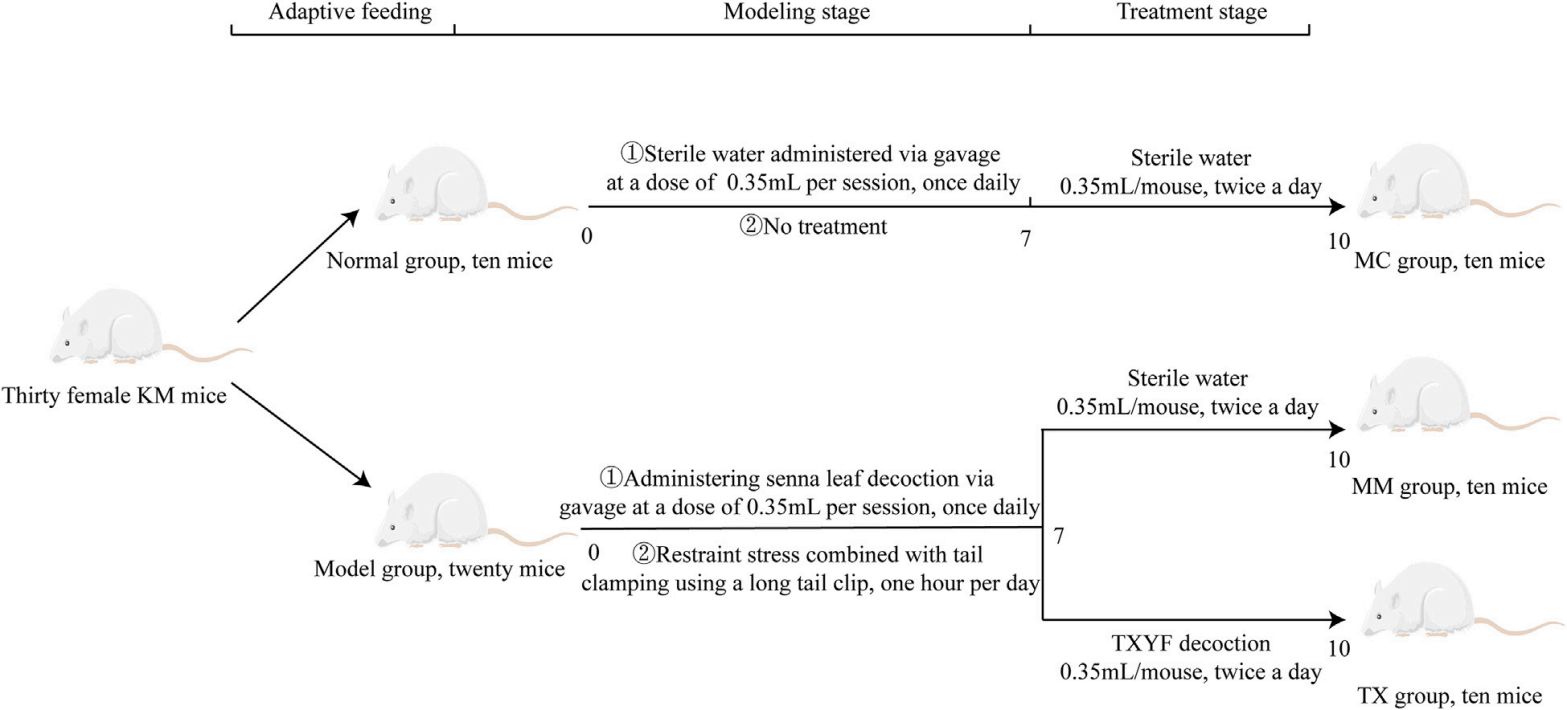
Experimental flow chart.

TCM treatment phase: Following successful model establishment, the mice in the MM group were randomly assigned to one of two subgroups: the Tongxie Yaofang (TX) group (n = 10) and the MM model control group (n = 10). According to the dose conversion method outlined in the Research Methods in Traditional Chinese Medicine Pharmacology [21, 22], the equivalent dose of the Tongxie Yaofang decoction for mice was determined to be 0.35 mL per administration, twice daily, for 3 consecutive days. The MC and MM groups received an equal volume of distilled water via gavage on the same schedule. The mice were fasted and not given water for 12 h before the operation. The treatment protocol is illustrated in Figure 1.

### Assessment of the treatment efficacy of IBS-D and Tongxie Yaofang

The clinical characteristics of IBS-D and relevant literature have been used to guide syndrome differentiation, with a focus on changes in fecal properties, aggressive or irritable behaviors, hyperactivity, dull coats, and decreased food and water intake. Mice treated with Tongxie Yaofang showed improvements in mood, coat condition, and fecal characteristics.

#### General conditions

Fecal samples were obtained on days 0, 2, 4, 6, 8, and 10. The number of defecations within 30 min was recorded for each mouse. Dietary intake and water consumption were monitored on days 1, 3, 5, 7, and 9. Body weight was recorded every other day throughout the experiment.

#### Measurement of food and water intake

At 9:00 a.m., preweighed feed was placed in each cage. After 48 h, the remaining feed was weighed to calculate the average food intake per mouse. Similarly, water consumption was measured over the same period to calculate the average intake per mouse [23].

#### Assessment of fecal moisture content and defecation frequency

Each mouse was situated in a sanitized, arid enclosure with unrestricted access to sustenance and hydration. After a duration of 30 min, the quantity of the fecal pellets was determined. Both single and paired pellets were regarded as singular fecal events. The frequency of defecations occurring within a 30-min interval was documented. The collected feces were desiccated at 110 °C until a consistent weight was achieved. All feces contaminated with pee were eliminated or absorbed to reduce mistakes [24].

#### Measurement of MTL, CRH and TBA in mice

Each mouse was weighed prior to blood collection and then euthanized by cervical dislocation. Blood samples were obtained and allowed to clot, and then centrifuged at 4 °C and 3000 revolutions per minute for 10 min. The serum was collected to analyze MTL, CRH, and TBA levels via ELISA kits. Following euthanasia, approximately 0.1 g of liver tissue was excised with sterile scissors, accurately weighed on an analytical balance, homogenized with saline and steel beads, and centrifuged under the same conditions. The supernatant was subsequently used to measure hepatic total bile acid content according to the ELISA kit protocol [25].

#### Organ index

After connective and adipose tissue were removed, the liver, spleen, and thymus were weighed [26]. Organ index (%) = organ index (g)/body weight (g) × 100%.

#### Colon mucosa collection

Colon segments from five mice per group were selected. The intestinal contents were removed, and the colon was opened longitudinally and rinsed with saline. The mucosa was scraped with a sterile coverslip, collected into 1.5 mL sterile tubes, snap-frozen in liquid nitrogen, and stored at −80 °C for sequencing [25].

#### Extraction of DNA, amplification of the 16S rRNA gene, and sequencing

Colon mucosal samples were analyzed by Shanghai Paisonno Biotechnology Co., Ltd.

(1) Genomic DNA was isolated via the OMEGA Soil DNA Kit (M5635-02). The quality and quantity of the products were evaluated via a NanoDrop spectrophotometer (Thermo Fisher Scientific, NC2000) and agarose gel electrophoresis (Beijing Liuyi, DYY-6C).
(2) PCR amplification was conducted via the forward primer 338F (5′-ACT​CCT​ACG​GGA​GGC​AGC​A-3′) and reverse primer 806R (5′-GGACTACHVGGGTWTCTAAT-3′) to target the bacterial 16S rRNA V3+V4 region.
(3) Product detection and recovery: PCR products were detected using a 1.2% agarose gel and subsequently purified using the AxyPrep PCR Recovery Kit (LS.3008-29).
(4) Fluorescence quantification: PCR products were measured via the Quant-iT PicoGreen dsDNA Assay Kit.
(5) Sequencing: Qualified libraries were generated via the Illumina TruSeq Nano DNA LT Kit and sequenced on an Illumina NovaSeq 6000 (PE250) [27]. The sequencing data of the mouse colon mucosal microbiome have been submitted to the National Center for Biotechnology Information database: PRJNA1256748.

#### Bioinformatics analysis

(1) Species taxonomy annotation [28]: The QIIME2 (2019.4) DADA2 pipeline was used for denoising and generating ASVs. Taxonomic classification was conducted via Greengenes 13.8^10^ and QIIME2’s classify-sklearn plugin^11^.
(2) Alpha diversity: Using QIIME2 and R (ggplot2), the alpha-rarefaction.qzv file was generated via the “qiime diversity alpha-rarefaction” command via QIIME2 (2019.4). The rarefaction curve (Chao1, Observed_species, Shannon, Simpson indices) was visualized by dragging the file into^12^.
(3) Beta diversity: Using the flattened ASV/OTU table, the weighted UniFrac distance matrix was calculated according to the “qiime diversity core-metrics-phylogenetic” command of the tree file, and PCoA was conducted. QZV files were visualized on^12^, and PCoA plots were generated in R.
(4) Species differences and marker species analysis [29]: LEfSe and random forest analyses were used to identify differentially abundant taxa and microbial biomarkers via QIIME2 (2019.4), Python, and R (ggtree).
(5) Functional prediction analysis: PICRUSt2 was used to predict the 16S rRNA gene sequence in the KEGG functional database^13^, and the primary, secondary, and tertiary functional abundances of the samples were determined.
(6) Correlation analysis: R and Cytoscape 3.9.1 were used to analyze correlations between the microbiota and physiological indicators such as CRH, MTL, TBA, and general phenotypic traits.

#### HE staining of mouse liver and spleen

The fixed tissues were dehydrated, embedded in paraffin, sectioned, dewaxed, and rehydrated. The sections were stained with hematoxylin for 3–5 min, differentiated, blued, and washed with running water. Following dehydration in a graded ethanol series, the sections were stained with eosin for 5 minutes. The slides were ultimately prepared with neutral glue, examined microscopically, and photos were captured and analyzed [30].

### Statistical methods

Statistical analyses were conducted using SPSS 25.0 software. Quantitative data that adhere to a normal distribution are represented as the mean ± standard deviation (x̅ ± SD). For comparisons between two groups exhibiting normally distributed data, the *t*-test was employed; conversely, if normality was not satisfied, the Mann–Whitney U test was utilized. One-way analysis of variance (ANOVA) was employed for comparisons among several groups, contingent upon the data meeting the criteria of normality and homogeneity of variance, followed by the least significant difference (LSD) *post hoc* test. If these assumptions were unmet, the Kruskal–Wallis test was performed. Categorical data were represented as percentages (%) and analyzed via the chi-square test. A significance level of α = 0.05 was established, with *p* < 0.05 deemed statistically significant.

## Results

### Investigation of the mechanism of Tongxie Yaofang in the treatment of IBS-D by network pharmacology

**FIGURE 2:**
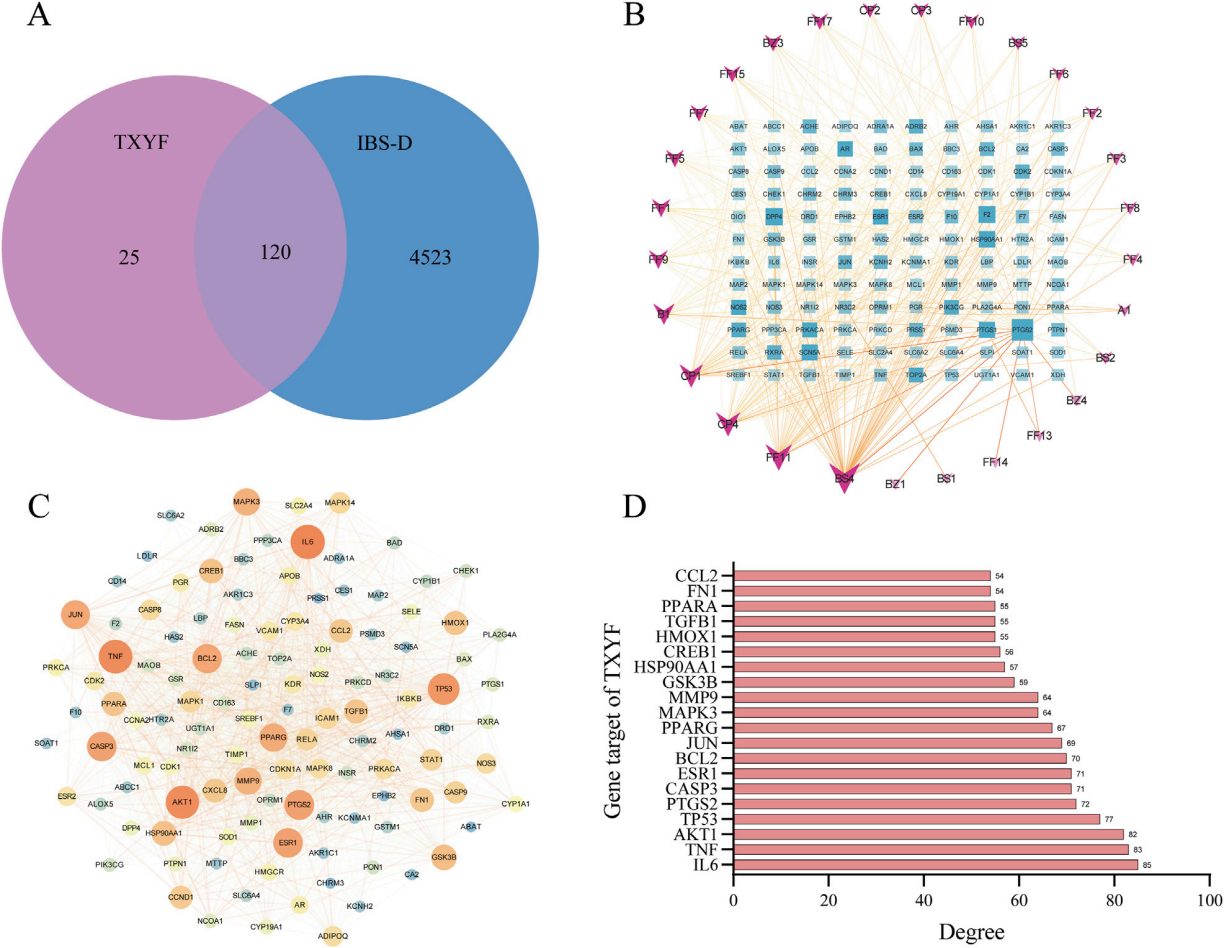
Network pharmacology assessment of Tongxie Yaofang. **(A)** A Venn diagram illustrating the intersection between the target genes of the active components of Tongxie Yaofang and the target genes associated with IBS-D; **(B)** The network was established with 28 active compounds and 120 gene targets of Tongxie Yaofang. The rectangular nodes signify gene targets, while the V-shaped nodes denote active substances. The depth of nodes exhibits a positive correlation with the degree value; **(C)** PPI network diagram of Tongxie Yaofang syndrome and IBS-D. Nodes signify targets, while edges denote protein-protein interactions. The color and size of nodes exhibit a positive correlation with the degree value, where a larger node size signifies greater importance; **(D)** The PPI network identified the top 20 gene targets of Tongxie Yaofang and IBS-D.

A total of 40 active ingredients in the four herbs of Tongxie Yaofang were identified through preliminary analysis (see Supplementary Table S1). By screening the TCMSP database, 145 compound-related targets were obtained. Additionally, 4,643 disease-related targets of IBS-D were identified via the use of the GeneCards and OMIM databases. As illustrated in Figure 2A, the Venn diagram displays the overlapping targets between the compounds and the disease, indicating that 28 of the 40 active ingredients (corresponding to 120 targets) were closely associated with the treatment of IBS-D.

To elucidate the multicomponent, multitarget synergistic effects of Tongxie Yaofang in the treatment of IBS-D and to further explore its underlying molecular mechanisms, a compound–target interaction network was constructed. As shown in Figure 2B, the network comprises 148 nodes and 354 edges. In this context, node size is positively correlated with node degree, reflecting the relative importance of a component or target within the network. Therefore, the components with the highest degrees—BS4 (kaempferol, degree = 52), FF11 (wogonin, degree = 40), CP4 (nobiletin, degree = 34), CP1 (naringenin, degree = 32), and B1 (beta-sitosterol, degree = 26)—were identified as the key active compounds in the treatment of IBS-D.

Figure 2C illustrates that protein–protein interaction (PPI) data sourced from the STRING database^2^ were imported into Cytoscape 3.9.1 to construct the PPI network of Tongxie Yaofang. The five targets with the highest degree values—IL-16 (degree = 85), TNF (degree = 83), AKT1 (degree = 82), TP53 (degree = 77), and PTGS2 (degree = 72)—were identified as core targets potentially mediating the therapeutic effects of Tongxie Yaofang in IBS-D patients, as illustrated in Figure 2D.

**FIGURE 3:**
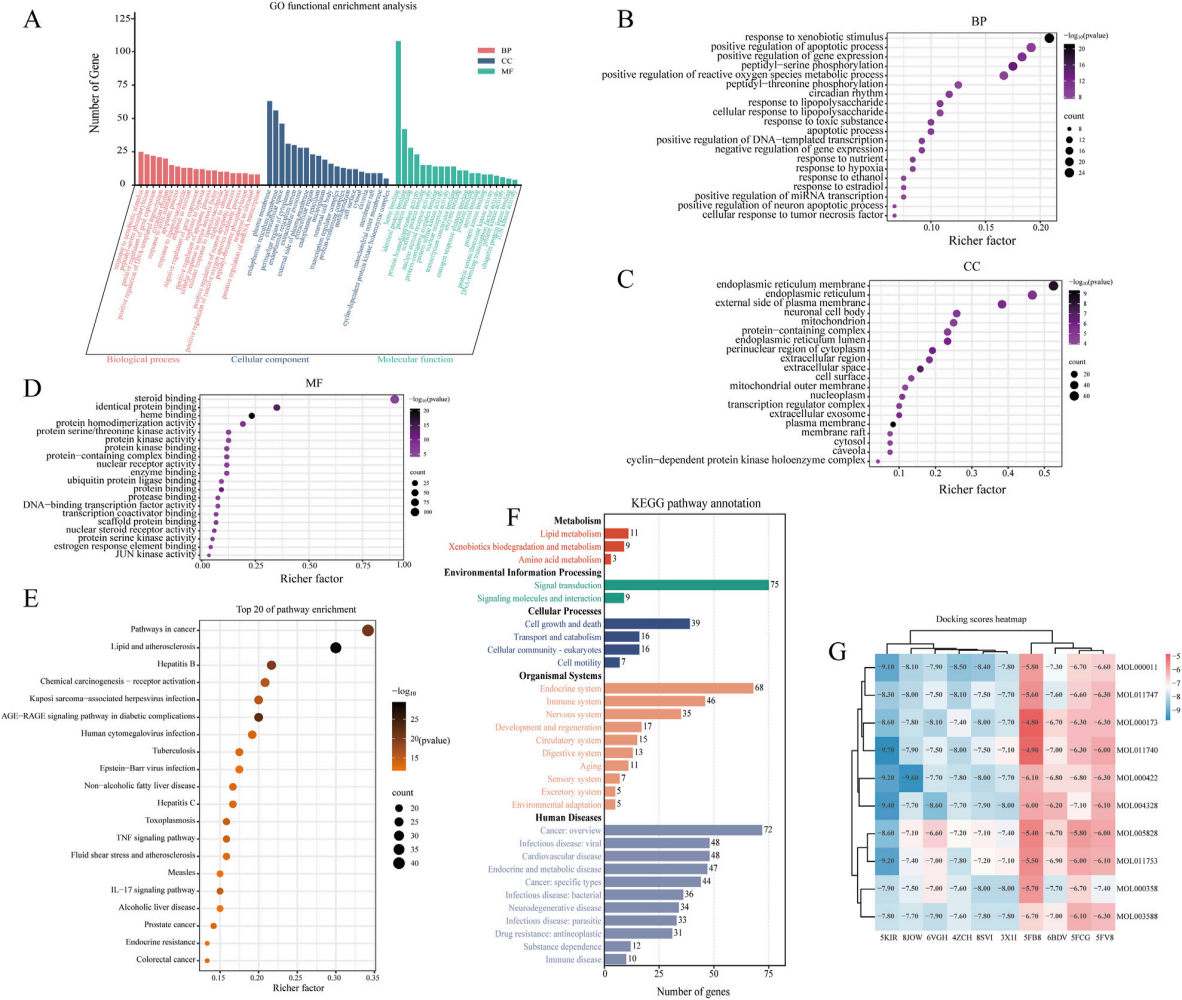
Network pharmacology analysis of Tongxie Yaofang. **(A)** GO functional enrichment analysis of the top 20 of the 120 gene targets; **(B)** Top 20 GO enrichments in BP; **(C)** Top 20 GO enrichments in CC; **(D)** Top 20 GO enrichments in MF. The X-axis represents the degree of gene enrichment, and the color of the dots represents the corresponding *P* value. Larger dots indicate that more genes are enriched; **(E)** KEGG top 20 enriched pathways. Larger dots indicate that more genes are enriched in the corresponding pathways; **(F)** KEGG pathway annotation. Bar graphs of different colors represent different aspects of the annotations of the 120 gene target pathways; **(G)** Heatmap of the molecular docking binding energy of TCM components and therapeutic targets.

GO enrichment analysis was performed to evaluate the overrepresentation of GO terms within the identified gene set, thereby elucidating the shared biological characteristics of these genes in terms of biological processes (BP), cellular components (CC), and molecular functions (MF), as illustrated in Figure 3A. Based on the criteria of *p* < 0.05 and a false discovery rate (FDR) < 0.05, a total of 216 biological processes, 38 cellular components, and 53 molecular functions were significantly enriched. As shown in Figures 3B–D, the top 20 significantly enriched terms indicated that Tongxie Yaofang may exert therapeutic effects by modulating pathways such as response to xenobiotic stimulus, positive regulation of apoptotic process, caveola, cytosol, enzyme binding, and identical protein binding.

Furthermore, KEGG pathway analysis (*p* < 0.05, FDR <0.05) revealed 166 enriched signaling pathways, suggesting that the therapeutic action of Tongxie Yaofang in IBS-D patients is closely associated with the AGE-RAGE, TNF, and IL-17 signaling pathways, as presented in Figure 3E. The functional categorization of these KEGG pathways revealed five major classifications: metabolism, environmental information processing, cellular processes, organismal systems, and human diseases. Among these pathways, those related to lipid metabolism, signal transduction, cell growth and death, the endocrine system, and cancer were highly enriched (Figure 3F).

Ultimately, to confirm the connection between the active molecules of Tongxie Yaofang and IBS-D-related targets, a multi-molecular docking study was conducted, and the resultant binding energies were displayed. The top three compound–target complexes exhibiting the most stable binding affinities were further subjected to molecular dynamics simulation, as shown in Figure 3G.

### Molecular docking-based investigation of the mechanism of action of Tongxie Yaofang in the treatment of IBS-D

To elucidate the potential molecular mechanisms underlying the therapeutic effects of Tongxie Yaofang on IBS-D, molecular docking was performed using the protein targets 5KIR and 8JOW, along with their respective active ligands: divaricatol (MOL011740), naringenin (MOL004328), and kaempferol (MOL000422). Binding energy, a critical parameter in molecular docking analysis, reflects the stability and affinity of the interaction between a ligand and its target protein. Generally, a more negative binding energy value indicates a stronger and more stable interaction [31].

The binding energy of 5KIR and divaricatol (MOL011740) was calculated to be −9.70 kcal/mol, stabilized by multiple interactions, including van der Waals forces, hydrogen bonds, carbon‒hydrogen bonds, and π‒sigma, alkyl, and π‒alkyl interactions. Five conventional hydrogen bonds were formed with the amino acid residues ARG, GLN, HIS, GLY, and TYR, with corresponding bond lengths of 2.32 Å, 2.54 Å, 1.92 Å, 2.75 Å, and 2.93 Å, respectively.

Similarly, 5KIR and naringenin (MOL004328) exhibited a binding energy of −9.40 kcal/mol, which was supported by van der Waals forces, hydrogen bonds, carbon‒hydrogen bonds, and alkyl and π‒alkyl interactions. Two conventional hydrogen bonds involving residues TYR and GLY, with bond lengths of 2.57 Å and 2.40 Å, respectively, were identified.

The docking of 8JOW and kaempferol (MOL000422) yielded a binding energy of −9.60 kcal/mol, which was also stabilized by van der Waals forces, hydrogen bonds, carbon‒hydrogen bonds, amide‒π stacking, and alkyl and π‒alkyl interactions. Four conventional hydrogen bonds were formed with GLN, TYR, THR, and SER, with bond lengths of 2.93 Å, 2.01 Å, 2.33 Å, and 2.83 Å, respectively.

**FIGURE 4:**
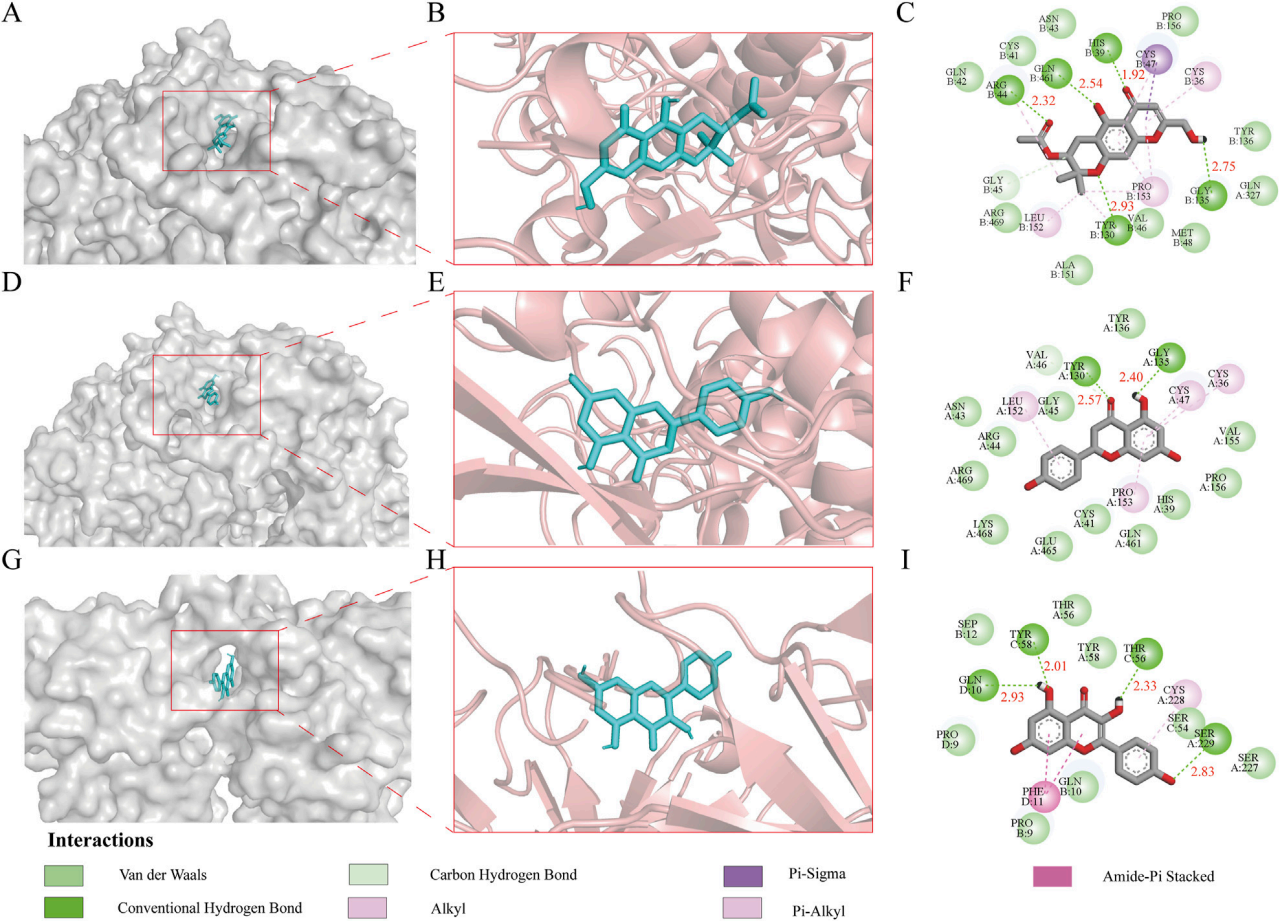
3D and 2D images of the molecular docking of the active ingredients of Tongxie Yaofang and the central target of IBS-D disease. **(A)** 3D surface structure of the 5KIR-divaricatol complex; **(B)** 3D internal structure of the 5KIR-divaricatol complex; **(C)** 2D structure of the 5KIR-divaricatol complex; **(D)** 3D surface structure of the 5KIR-naringenin complex; **(E)** 3D internal structure of the 5KIR-naringenin complex; **(F)** 2D structure of the 5KIR-naringenin complex; **(G)** 3D surface structure of the 8JOW-kaempferol complex; **(H)** 3D internal structure of the 8JOW-kaempferol complex; **(I)** 2D structure of the 8JOW-kaempferol complex.

These results, illustrated in Figure 4, demonstrate that the active compounds of Tongxie Yaofang exhibit strong and stable binding affinities with key IBS-D-associated targets, suggesting a robust pharmacodynamic basis for their therapeutic potential.

### Molecular dynamics-based study of the mechanism of action of Tongxie Yaofang in the treatment of IBS-D

#### MD simulation of the 5KIR-divaricatol complex

**FIGURE 5:**
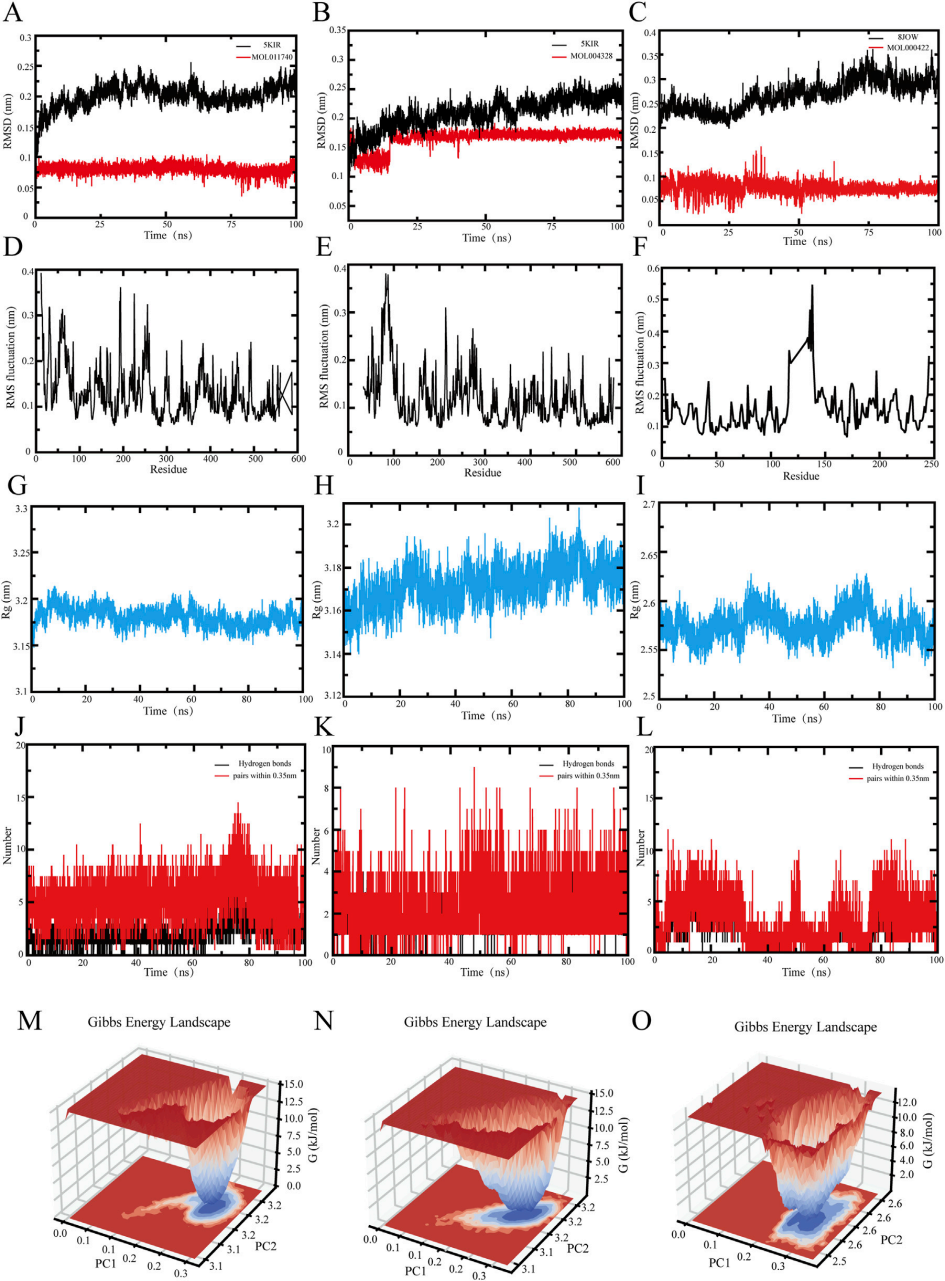
Molecular dynamics modeling of the active components of Tongxie Yaofang and the primary target of IBS-D. **(A–C)** Root mean square deviation (RMSD) of the protein and ligand; **(D–F)** Root mean square fluctuation (RMSF) of the protein; **(G–I)** Radius of gyration of the protein backbone (Rg); **(J–L)** Number of hydrogen bonds between the protein and ligand complex; **(M–O)** Gibbs free energy profile of the protein and ligand complex.

The RMSD curve reflects the structural deviation of the system from its initial conformation over time and is a key indicator of the overall flexibility and stability of the simulated system [32]. Higher RMSD values indicate stronger fluctuations between the protein and the small molecule. As shown in Figure 5A, the 5KIR-divaricator (MOL011740) complex remains relatively stable over the 0–100 ns simulation period, with the RMSD of 5KIR fluctuating between 0.1 and 0.25 nm. This finding suggests that while local flexibility adjustments occur, overall protein folding remains stable.

The RMSF describes the fluctuation amplitude of individual atomic positions, allowing insight into residue-level flexibility and the influence of ligand binding [33]. Upon ligand binding, protein flexibility often decreases, which helps to stabilize the active site. As shown in Figure 5D, residues 0–250 presented RMSF values above 0.2 nm, whereas residues 250 – 600 maintained lower values, indicating that the core structure of 5KIR is rigid, with increased stability following ligand interaction.

The Rg, which represents the average distance of atoms from the system’s center of mass, reflects protein compactness and aggregation [34]. Larger Rg values indicate a looser, less stable structure. As shown in Figure 5G, the Rg of the 5KIR backbone fluctuates consistently between 3.15 and 3.20 nm over the simulation period, indicating a compact and dynamically stable conformation.

Hydrogen bonding is the strongest form of noncovalent interaction and contributes significantly to ligand–receptor binding stability. Figure 5J shows that the 5KIR-divaricatol complex consistently maintains approximately five hydrogen bonds, suggesting a stable interface and a resilient hydrogen bond network.

The Gibbs free energy landscape constructed using the RMSD and Rg provides an intuitive visualization of the system’s conformational stability and transition states [35]. In Figure 5M, the blue region indicates a low-energy, high-probability conformation cluster, indicating a stable binding state. The red area marks higher-energy transition states. When PC1 = 0.2 – 0.3 and PC2 = 3.2, the 5KIR–divaricatol complex exists in a relatively stable conformation.

#### MD simulation of the 5KIR-naringenin complex

Figure 5B shows the RMSD curve of the 5KIR–naringenin (MOL004328) complex. The system remains stable throughout the 0 – 100 ns simulation, with minor fluctuations in the ligand at 20 ns, which stabilize afterward. The RMSD range of 0.1–0.175 nm indicates strong structural conservation.

According to Figure 5E, the RMSF of residues 50 – 250 exceeds 0.2 nm, whereas that of residues 250 – 600 is mostly less than 0.2 nm. This finding suggests that the core region of 5KIR retains rigidity upon ligand binding, with the termini showing greater flexibility.

The Rg results in Figure 5H show a stable fluctuation range of 3.14–3.21 nm, indicating that the complex has reached stable and compact structural equilibrium.

As shown in Figure 5K, the number of hydrogen bonds between 5KIR and naringenin remains approximately two throughout the simulation, which is fewer than that in the 5KIR–divaricatol complex, possibly owing to competition with solvent molecules, side-chain rotation, or conformational drift.

The Gibbs free energy surface in Figure 5N indicates a dominant, stable conformation when PC1 = 0.2 – 0.3 and PC2 = 3.1 – 3.2, confirming that the 5KIR–naringenin complex maintains conformational stability during the simulation.

#### MD simulation of the 8JOW-kaempferol complex

The RMSD curve in Figure 5C shows that the 8JOW–kaempferol (MOL000422) complex remains stable throughout the 0 – 100 ns simulation. The protein RMSD stabilized after a minor fluctuation at 20 ns, and the ligand RMSD stabilized after 30 ns, remaining within a fluctuation range of 0–0.15 nm. This suggests that the complex structure remains close to its initial conformation.

As shown in Figure 5F, residues 100 – 150 exhibit RMSF values above 0.3 nm, whereas residues 0 – 100 and 150 – 600 remain below 0.25 nm. This finding indicates that, apart from some flexible regions—possibly loops or functional domains—the 8JOW protein maintains rigidity. The flexible regions may play roles in ligand accommodation and allosteric regulation.

The Rg values in Figure 5I remain within 2.525–2.625 nm, indicating a stable and compact conformation throughout the simulation period.

According to Figure 5L, the complex consistently maintains approximately four hydrogen bonds, which exceeds the number of hydrogen bonds in the 5KIR–divaricatol complex, indicating relatively strong structural stability. The decrease in the number of hydrogen bonds from 30 to 80 ns suggests transient conformational changes or ligand displacement from the binding pocket.

In Figure 5O, the Gibbs free energy landscape indicates that the 8JOW–kaempferol complex adopts a stable conformation when PC1 = 0.2 – 0.3 and PC2 = 2.5 – 2.6, with low-energy clusters representing preferred binding states.

### Effects of Tongxie Yaofang on the macroscopic signs of diarrhea caused by liver hyperactivity with spleen deficiency pattern in mice

**FIGURE 6:**
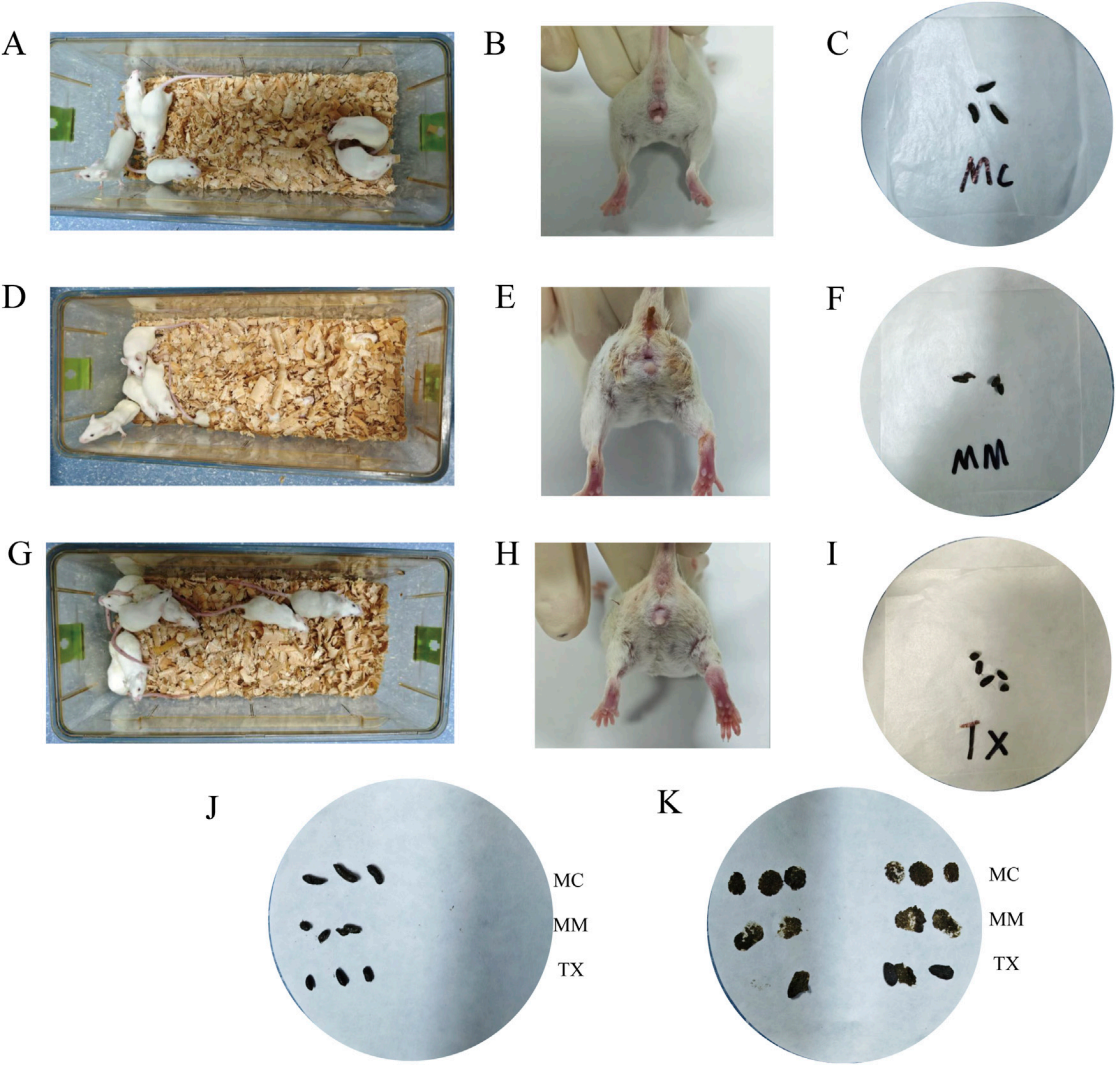
Impact of Tongxie Yaofang on the overall behavior of mice exhibiting diarrhea due to liver hyperactivity with spleen deficiency pattern. **(A)** Behavior and activity status of mice in the MC group; **(B)** Experimental and perianal conditions of mice in the MC group; **(C)** Feces of mice in the MC group; **(D)** Behavior and activity status of mice in the MM group; **(E)** Anal and perianal conditions of mice in the MM group; **(F)** Feces of mice in the MM group; **(G)** Behavior and activity status of mice in the TX group; **(H)** Anal and perianal conditions of mice in the TX group; **(I)** Feces of mice in the TX group; **(J,K)** Fecal attributes and moisture content of the mice.

As depicted in Figures 6A–C, the mice in the MC group presented a healthy appearance: an alert posture, glossy smooth fur, well‐formed black feces, a clean perianal area, and dry bedding. In contrast, the MM group (Figures 6D–F) presented clinical signs of illness, including hunching, irritability, a tendency to cluster and burrow, dull fur, yellowish loose and sticky stools, severe perianal soiling, and damp bedding. Following the administration of Tongxie Yaofang (Figures 6G–I), the treated mice showed marked improvement: hunching and excessive jumping were alleviated, appetite increased, clustering behavior decreased, fecal color reverted to black, and stool consistency normalized from watery to formed and dry.

As illustrated in Figures 6J,K, fecal samples from MM group mice appeared yellowish, and when they were gently blotted with filter paper, the resulting smear was notably more diffuse than that observed in the MC and TX groups. These findings indicate that Tongxie Yaofang treatment partially restores normal stool consistency and reduces perianal contamination in the liver hyperactivity with spleen deficiency pattern diarrhea model.

**FIGURE 7:**
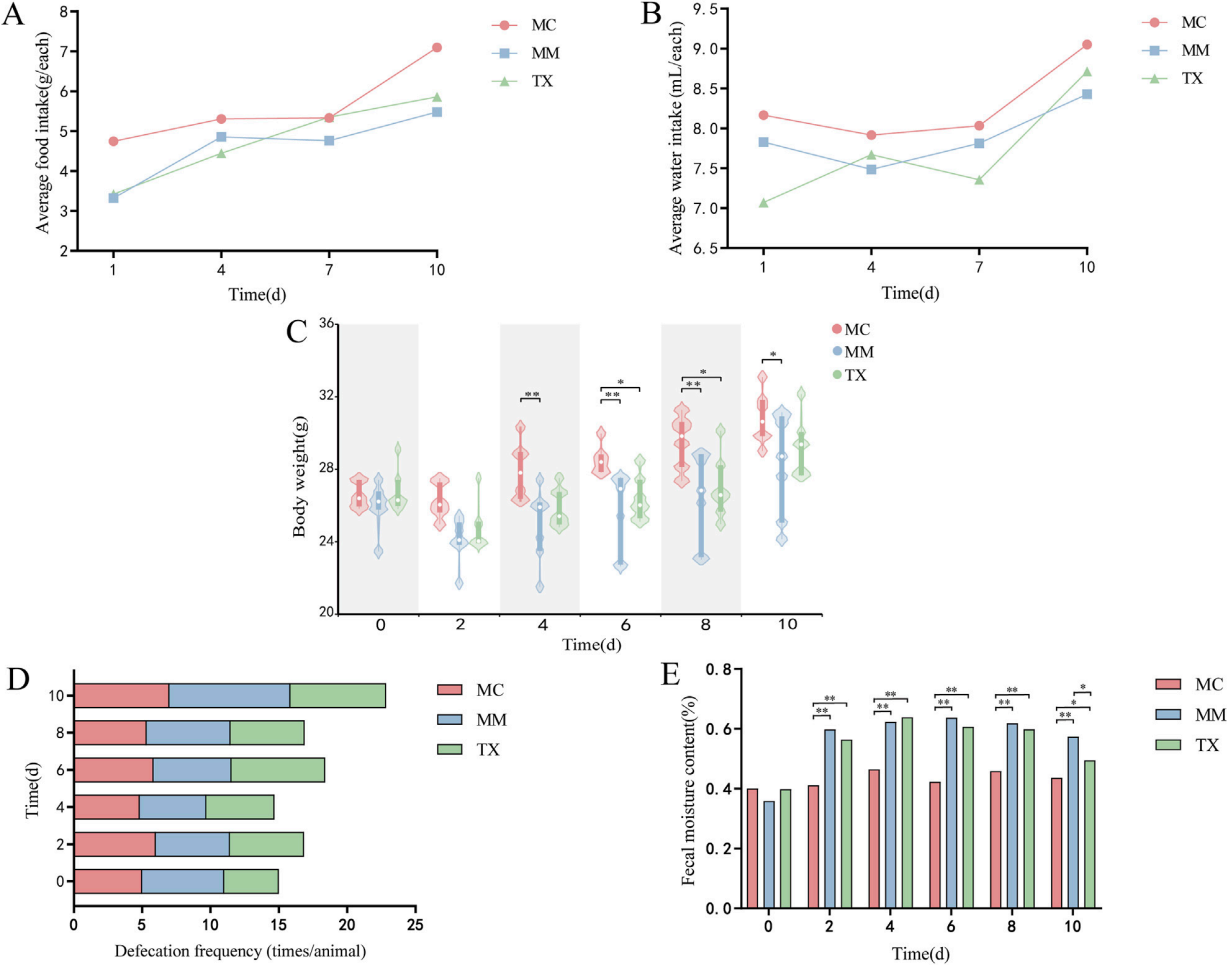
General characteristics of mice with diarrhea caused by liver hyperactivity with spleen deficiency pattern treated with Tongxie Yaofang. **(A)** Average food intake; **(B)** Average water intake; **(C)** Body weight; **(D)** Defecation frequency; **(E)** Fecal moisture content. *: *p* < 0.05, **: *p* < 0.01.

Figures 7A,B illustrate that, throughout the modeling period, both food and water intake in the MM and TX groups were inferior to that of the MC group. Following the initiation of Tongxie Yaofang treatment on day 8, water intake in the TX group increased significantly, whereas that in both the MC and MM groups gradually increased. As shown in Figure 7C, body weight increased across all groups. However, after *Senna alexandrina* Mill. administration, the baseline weights of the MM and TX groups decreased. By day 3 of treatment, the weight difference between MC and MM mice reached significance (*p* < 0.01). Postmodeling, MC mice weighed significantly more than MM (*p* < 0.01) and TX (*p* < 0.05) mice did. From day 8, mice in the TX group received intragastric administration of Tongxie Yaofang, which resulted in a marked increase in body weight. After treatment ended on day 10, the body weight of MC mice was significantly different from that of MM mice (*p* < 0.05), whereas no significant difference was observed between the TX and MM groups. The defecation frequency (Figure 7D) was similar across all groups at baseline but increased progressively in the MM group during modeling. Following treatment, the defecation frequency of the TX group returned to levels comparable with those of the MC group. Fecal moisture content—a key indicator of diarrhea — was unchanged at baseline (Figure 7E). Compared with the MC group, the MM and TX groups presented significantly greater moisture contents (*p* < 0.01) throughout the model. After 3 days of treatment, the difference between MC and TX diminished (*p* < 0.05), and MM versus TX also differed (*p* < 0.05), confirming the successful induction of diarrhea, in which Tongxie Yaofang improved the diet, water intake, body weight loss, increased defecation, and elevated fecal moisture.

### Effects of Tongxie Yaofang on immune and serum indices in diarrheal mice with liver hyperactivity with a spleen deficiency pattern

**FIGURE 8:**
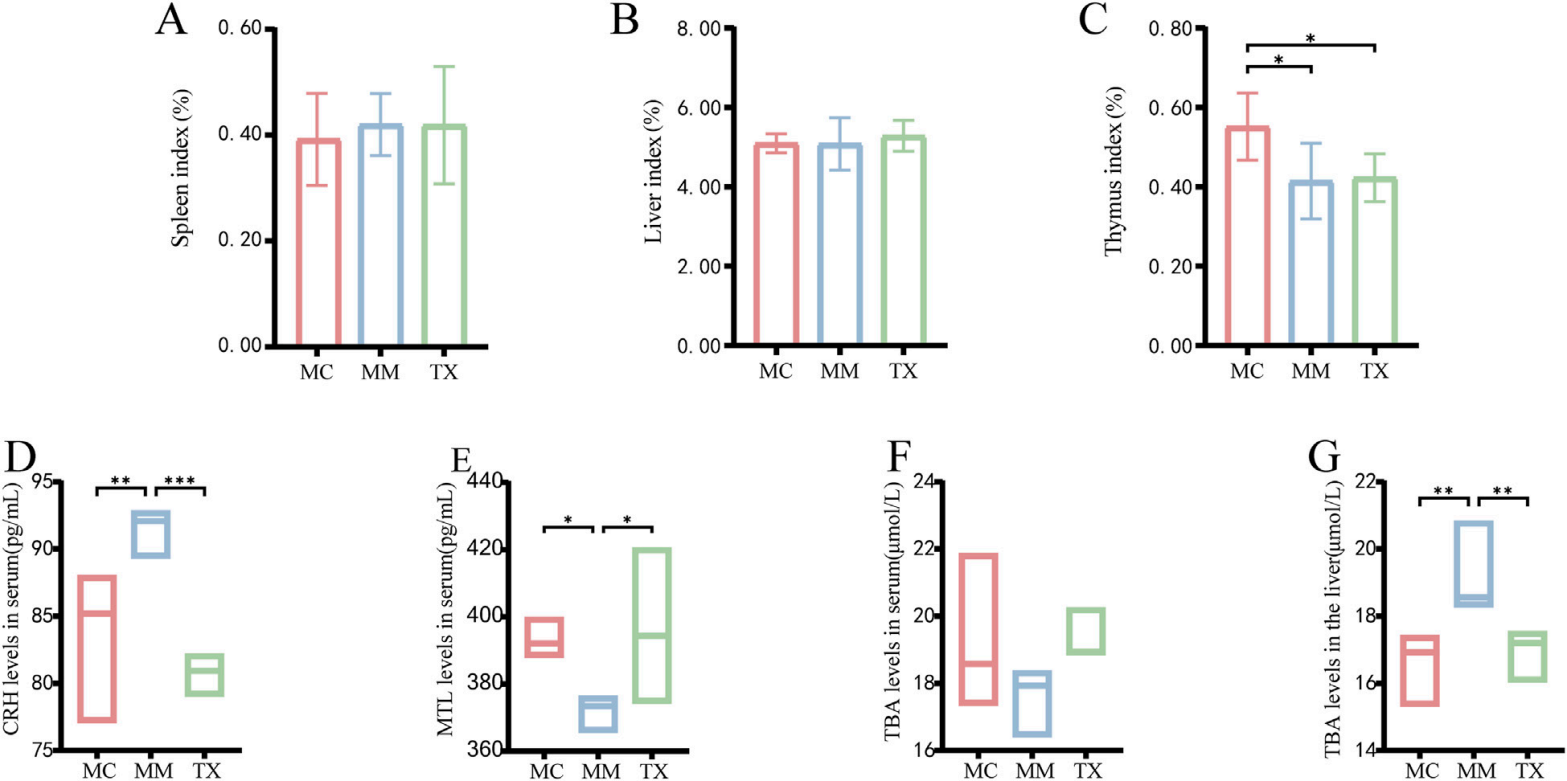
Effects of Tongxie Yaofang on organ indexes, MTL, CRH and TBA in mice with diarrhea caused by liver hyperactivity with spleen deficiency pattern. **(A)** Spleen index; **(B)** Liver index; **(C)** Thymus index; **(D)** Serum CRH content; **(E)** Serum MTL content; **(F)** Serum TBA content; **(G)** Liver TBA content. *: *p* < 0.05, **: *p* < 0.01, ***: *p* < 0.001.

As shown in Figure 8A, the spleen index tended to increase following *Senna alexandrina* Mill.- and restraint stress modeling but did not differ significantly from that of MC mice (*p* > 0.05). After 3 days of Tongxie Yaofang treatment, the spleen index in the TX group decreased slightly. Figure 8B shows that the liver index remained unchanged in both the MC and MM groups post-modeling (*p* > 0.05), whereas the TX group displayed a modest upwards trend after treatment. As shown in Figure 8C, the thymus index was significantly lower in MM and TX mice than in MC mice (*p* < 0.01); Tongxie Yaofang administration produced a slight, nonsignificant recovery.

Figure 8D shows that the model induced a significant increase in the serum CRH concentration in MM mice compared with that in MC mice (*p* < 0.01). Treatment with Tongxie Yaofang significantly reduced CRH below MC levels (*p* < 0.001). As shown in Figure 8E, the serum MTL decreased in MM mice relative to MC mice (*p* < 0.05) and was restored to baseline following TX treatment. Figures 8F,G illustrate the changes in TBA levels. In the model group, the serum TBA level decreased, which normalized after Tongxie Yaofang administration, whereas the liver TBA level increased significantly in the MM group (*p* < 0.01) and returned to MC levels in the TX group.

### Histopathological evaluation of the liver and spleen in diarrheal mice with liver hyperactivity with spleen deficiency pattern

**FIGURE 9:**
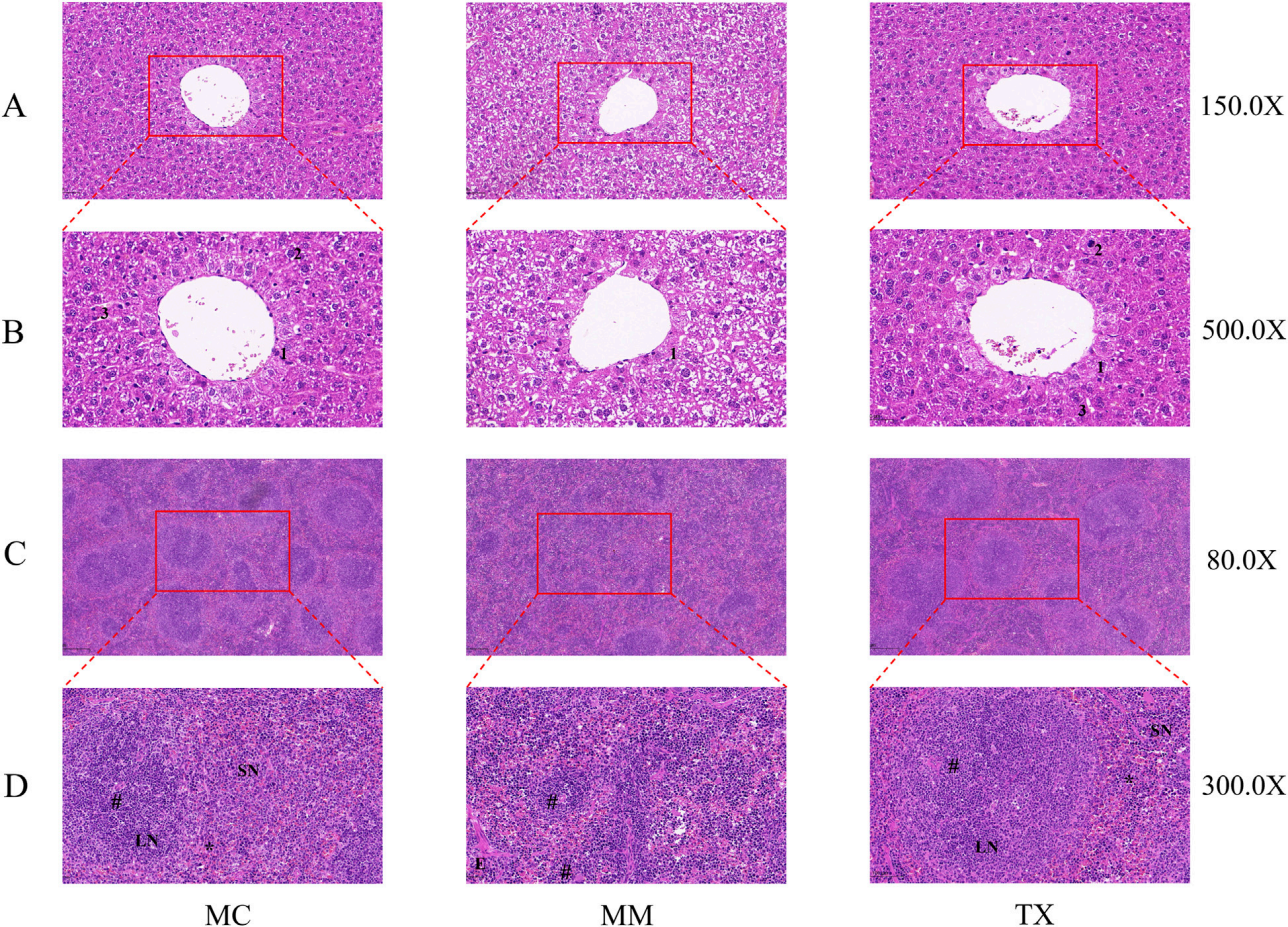
Impact of Tongxie Yaofang on pathological specimens of hepatic and splenic tissues in mice exhibiting diarrhea attributed to liver hyperactivity with spleen deficiency pattern. **(A)** ×150 magnification of liver tissue; **(B)** ×500 magnification of liver tissue; **(C)** ×150 magnification of spleen tissue; **(D)** ×500 magnification of spleen tissue. 1: Hepatic epithelial cells; 2: nuclei; 3: hepatic sinusoids; LN: white pulp; SN: red pulp; #: central artery; *: marginal sinus; E: splenic trabeculae.

As shown in Figure 9, HE staining of MC-group livers revealed an intact lobular architecture, clear and patent hepatic sinusoids, radially arranged polygonal hepatocytes with uniform cytoplasmic eosinophilia, distinct nuclei, and no hepatocellular swelling, degeneration, or inflammatory infiltration. In contrast, MM-group livers presented a disrupted lobular structure, hepatocellular swelling and rupture, and marked inflammatory cell infiltration. The TX group notably attenuated hepatocyte damage and reduced inflammatory infiltration, restoring near-normal lobular organization.

In the MC group spleens, the white and red pulp areas were well demarcated, with dense parenchymal organization, distinct splenic corpuscles exhibiting prominent germinal centers, and balanced white-to-red pulp proportions. MM mice displayed substantial pathology, including marked white pulp atrophy, disrupted architecture with white pulp reduced to scattered cords or small clusters, and blurred white‒red pulp boundaries. Following Tongxie Yaofang administration, the spleens from the TX group presented improved white pulp preservation, clearer demarcation between white and red pulp, and restoration of the normal splenic architecture.

### Effects of Tongxie Yaofang on colonic mucosal microbial richness and diversity in mice with diarrhea caused by liver hyperactivity with spleen deficiency pattern

**FIGURE 10:**
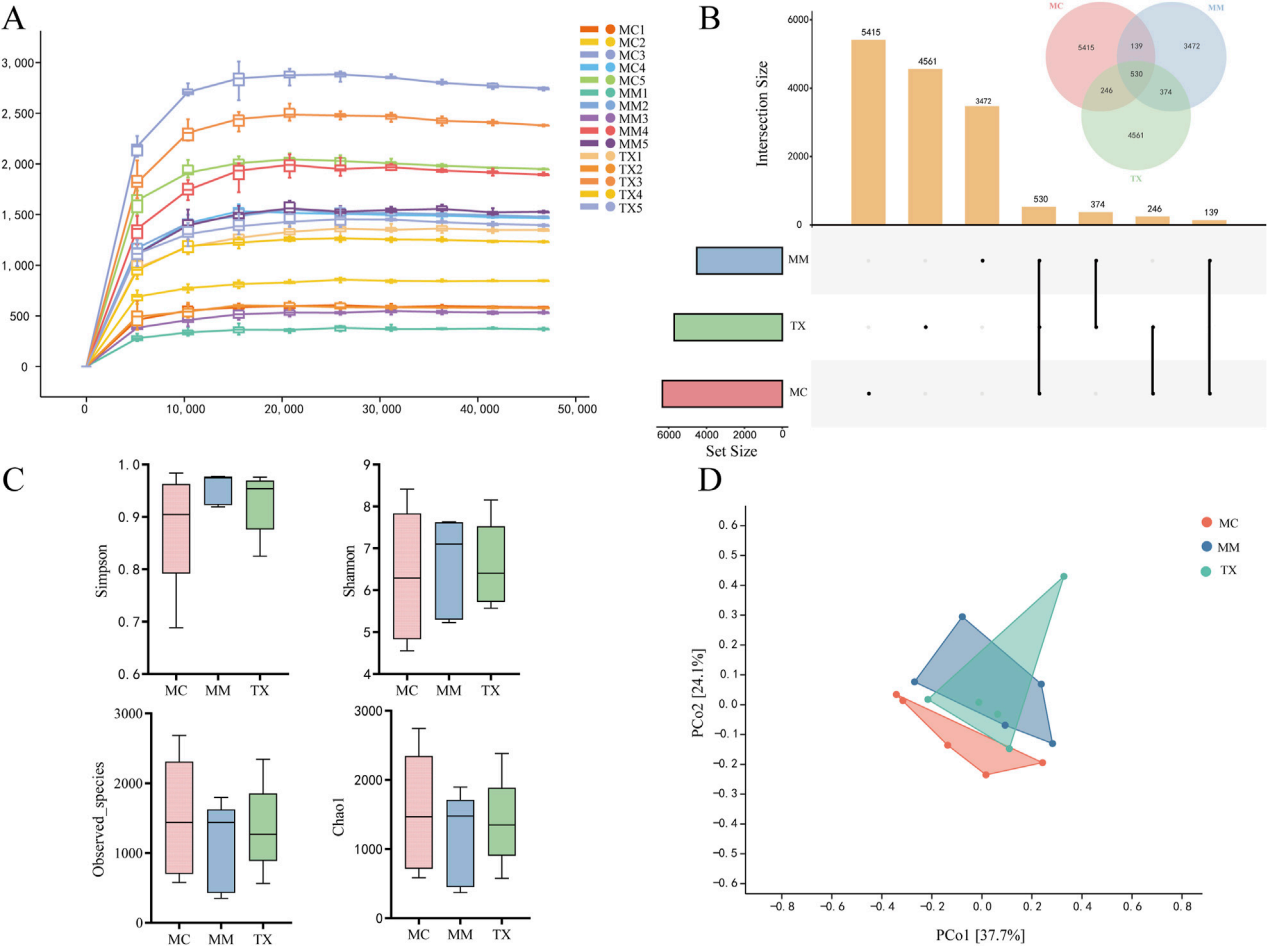
Analysis of the structure of the colonic mucosal microbiota. **(A)** Rarefaction curve; **(B)** ASV/OUT UpSet plot; **(C)** Alpha diversity indices, namely, Simpson, Shannon, Observed_species and Chao1 indices; **(D)** Distance matrix and PcoA.

The rarefaction curve illustrates how α diversity changes with sequencing depth, and its plateau indicates that additional sequencing yields few new species. As shown in Figure 10A, the curves for the MC, MM, and TX groups all levelled off, demonstrating that sequencing depth adequately captured the vast majority of the microbial taxa. The ASV/OTU UpSet plot (Figure 10B) quantified unique and shared taxa: the MC group contained 6330 ASVs (5415 unique), the MM group 4515 ASVs (3472 unique), and the TX group 5711 ASVs (4561 unique).

α-diversity indices assess species richness and evenness. The Chao1 and Observed_species indices quantify richness, while the Shannon and Simpson indices assess diversity. Figure 10C illustrates that there were no significant changes in Chao1 (*p* = 0.76) or Observed_species (*p* = 0.68) across the groups. The MM and TX groups presented nonsignificant upwards trends in the Simpson index (*p* = 0.44), and the Shannon index of the TX group returned to MC levels without reaching significance (*p* = 0.89). These results indicate that the model perturbed microbial richness and diversity and that Tongxie Yaofang partially restored these parameters.

β-diversity analysis via principal coordinate analysis (PCoA) visualizes intersample differences. Figure 10D shows that PCo1 and PCo2 explain 37.7% and 24.1% of the variance, respectively. The MC and MM groups had separate clusters, indicating that modeling substantially modified the mucosal microbiota composition. The TX group exhibited partial overlap with the MM group while leaning towards the MC cluster, suggesting that Tongxie Yaofang facilitated the regeneration of the colonic mucosal microbial population.

### Impact of Tongxie Yaofang on the predominant colonic mucosal microbiota in diarrheal mice with diarrhea caused by liver hyperactivity with spleen deficiency pattern

**FIGURE 11:**
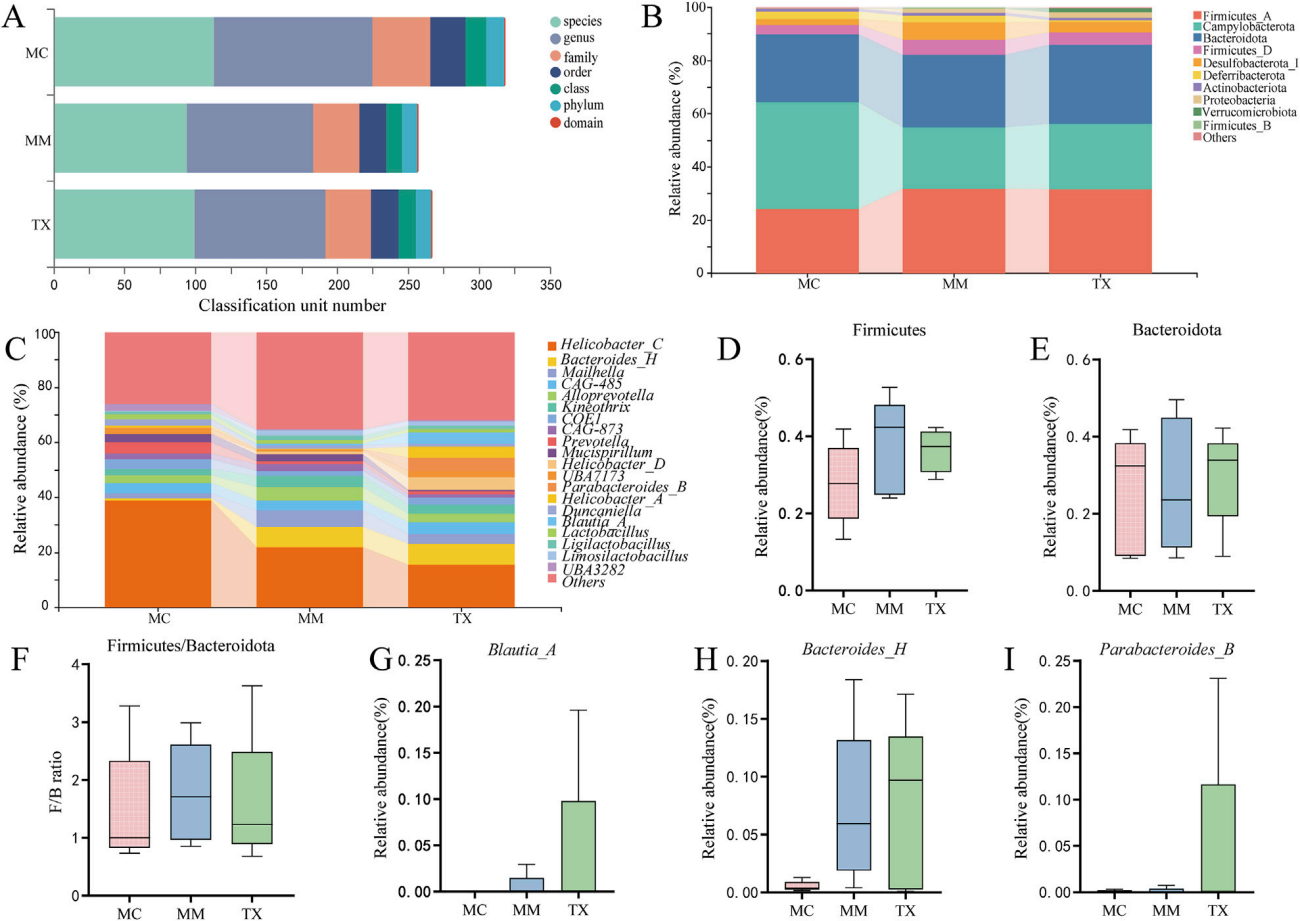
Effects of Tongxie Yaofang on the dominant microbial community in the colon mucosa of mice with diarrhea caused by liver hyperactivity with spleen deficiency pattern. **(A)** Multiaxis bubble chart; **(B)** Gate horizontal bar chart; **(C)** Genus horizontal bar chart; **(D,E)** Dominant gate; **(F)** F/B ratio; **(G–I)** Dominant genus.

Figure 11A illustrates the relative abundances of the top 10 phyla and top 20 genera in the colonic mucosa prior to and following modeling and treatment. At the phylum level (Figure 11B), Firmicutes, Campylobacterota, Bacteroidota, and Desulfobacterota_I predominated. Compared with the MM group, the TX group exhibited an increase in the abundance of Campylobacterota and Bacteroidota, while demonstrating a decrease in Deferribacterota abundance. The Firmicutes and Desulfobacterota_I levels in the TX group closely resembled those in the MC group. The modeling elevated the Firmicutes-to-Bacteroidota (F/B) ratio in MM, which was diminished in the TX group, while the change was not statistically significant (*p* > 0.05) (Figures 11D–F).

At the genus level (Figure 11C), *Blautia_A*, *Parabacteroides_B*, and *Bacteroides_H* were the most abundant. As shown in Figures 11G–I, *Blautia_A* comprised 3.92% of the TX microbiota, which was significantly greater than that reported for MM (0.59%) and MC (0%). The *Bacteroides_H* in TX (7.43%) closely matched that in MM (7.22%) and far exceeded that in MC (0.53%). *Parabacteroides_B* rose to 4.69% in TX, paralleling the trend observed for *Blautia_A*, although the intergroup differences were not statistically significant (*p* > 0.05).

These shifts indicate that Tongxie Yaofang partially restored the dominant microbial community structure toward that of healthy controls.

### Effect of Tongxie Yaofang on the characteristic colonic mucosal microbiota in diarrheal mice with liver hyperactivity with spleen deficiency pattern

**FIGURE 12:**
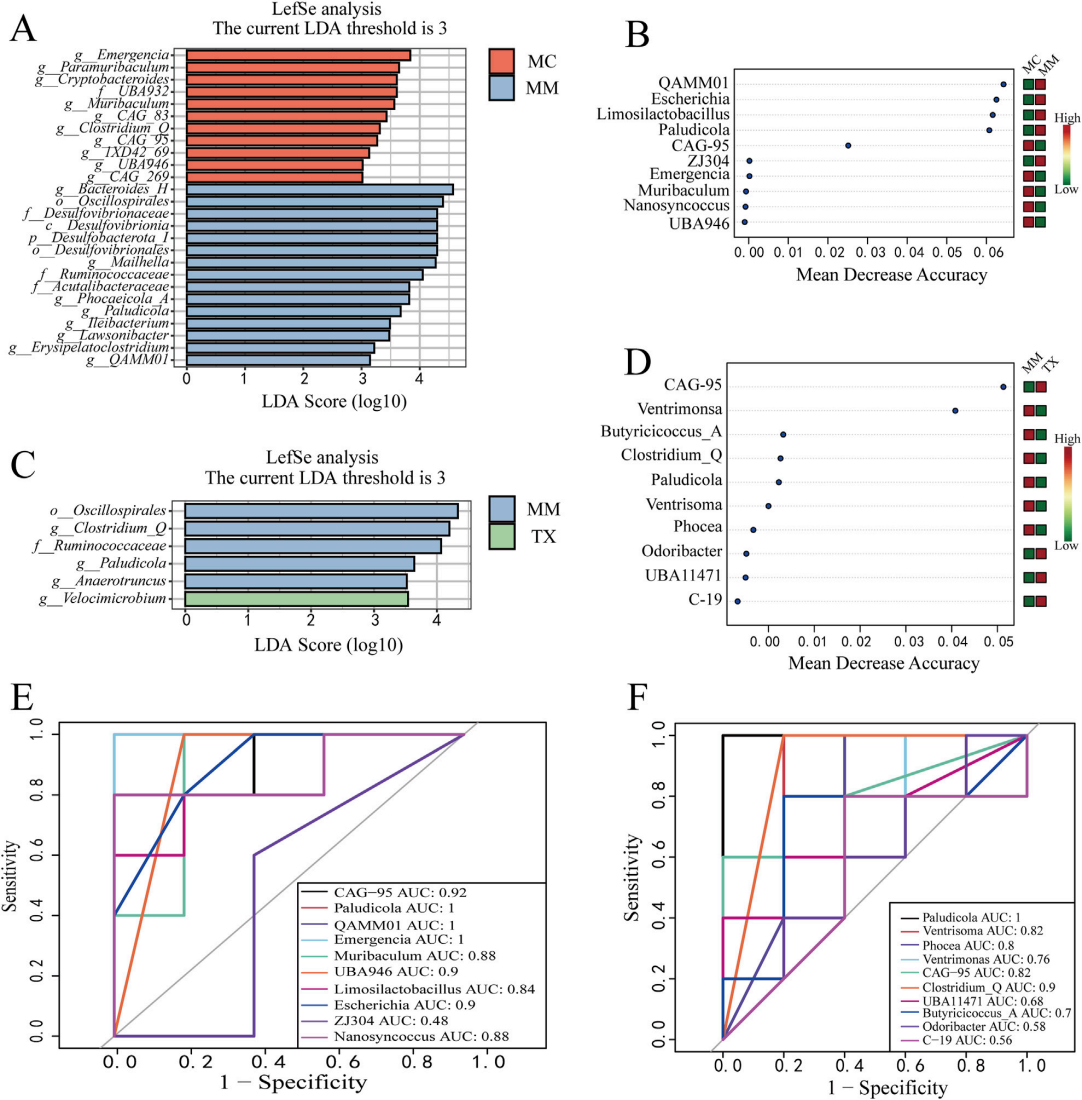
Effects of Tongxie Yaofang on characteristic microorganisms of the colon mucosa in mice with diarrhea caused by liver hyperactivity with spleen deficiency pattern. **(A,C)** LDA plots; **(B,D)** Random forest plots at the genus level; **(E,F)** ROC curves at the genus level between groups. E: MC *VS* MM; F: MM *VS* TX.

Linear discriminant analysis effect size (LEfSe) was conducted to identify genera with significantly different abundances among groups (LDA score >3). Comparing MC versus MM (Figure 12A), ten bacterial genera were enriched in the MC group: *Emergencia* (LDA = 3.8364, *p* = 0.0054), *Paramuribaculum* (LDA = 3.6441, *p* = 0.0472), *Cryptobacteroides* (LDA = 3.6039, *p* = 0.0465), *Muribaculum* (LDA = 3.5622, *p* = 0.0283), *Clostridium_Q* (LDA = 3.3128, *p* = 0.0163), *CAG_83* (LDA = 3.4289, *p* = 0.0163), *CAG_95* (LDA = 3.2678, *p* = 0.0264), *1XD42_69* (LDA = 3.1316, *p* = 0.0186), *UBA946* (LDA = 3.0177, *p* = 0.0186), and *CAG_269* (LDA = 3.0150, *p* = 0.0343). Whereas eight bacterial genera were enriched in the MM group: *Bacteroides_H* (LDA = 4.5705, *p* = 0.0283), *Mailhella* (LDA = 4.2726, *p* = 0.0472), *Phocaeicola_A* (LDA = 3.8170, *p* = 0.0472), *Paludicola* (LDA = 3.6712, *p* = 0.0090), *Ileibacterium* (LDA = 3.4858, *p* = 0.0343), *Lawsonibacter* (LDA = 3.4750, *p* = 0.0472), *Erysipelatoclostridium* (LDA = 3.2184, *p* = 0.0132), and *QAMM01* (LDA = 3.1437, *p* = 0.0053). When MM was compared with TX (Figure 12C), *Clostridium_Q* (LDA = 4.1977, *p* = 0.0186), *Paludicola* (LDA = 3.6399, *p* = 0.0090), and *Anaerotruncus* (LDA = 3.5217, *p* = 0.0472) remained enriched in MM, whereas *Velocimicrobium* (LDA = 3.5415, *p* = 0.0186) was the sole genus enriched in the TX group.

A random forest model ranked the top 10 discriminatory genera across MC, MM, and TX (Figures 12B,D). In the MC vs. MM comparison, *Phocaeicola_A* ***,*** *QAMM01*, *Escherichia*, and *Limosilactobacillus* exhibited importance >0.04 and higher abundance in MM. In MM vs. TX, *CAG_95* and *Ventrisoma* scored >0.04, with *CAG_95* enriched in TX and *Ventrisoma* enriched in MM.

Receiver operating characteristic (ROC) analysis (AUC >0.8) was used to assess diagnostic performance. In MC and MM groups (Figure 12E), the genera with an AUC >0.8 included *CAG_95* (AUC = 0.92), *Paludicola* (AUC = 1.00), *QAMM01* (AUC = 1.00), *Emergencia* (AUC = 1.00), *Muribaculum* (AUC = 0.88), *UBA946* (AUC = 0.88), *Limosilactobacillus* (AUC = 0.84), *Escherichia* (AUC = 0.90), and *Nanosyncoccus* (AUC = 0.88). In the MM and TX groups (Figure 12F), *Paludicola* (AUC = 1.00), *Ventrisoma* (AUC = 0.82), *Phocea* (AUC = 0.80), *CAG_95* (AUC = 0.82), and *Clostridium_Q* (AUC = 0.90) met the AUC criterion.

In summary, *CAG_95*, *Paludicola*, *QAMM01*, *Emergencia*, *Muribaculum*, and *UBA946* are potential diagnostic markers for the diarrhea model, whereas *Paludicola*, *Ventrisoma*, *CAG_95*, and *Clostridium_Q* emerge as candidate therapeutic biomarkers following Tongxie Yaofang intervention.

### Effect of Tongxie Yaofang on the function of colonic mucosal microorganisms in mice with liver hyperactivity with spleen deficiency pattern

**FIGURE 13:**
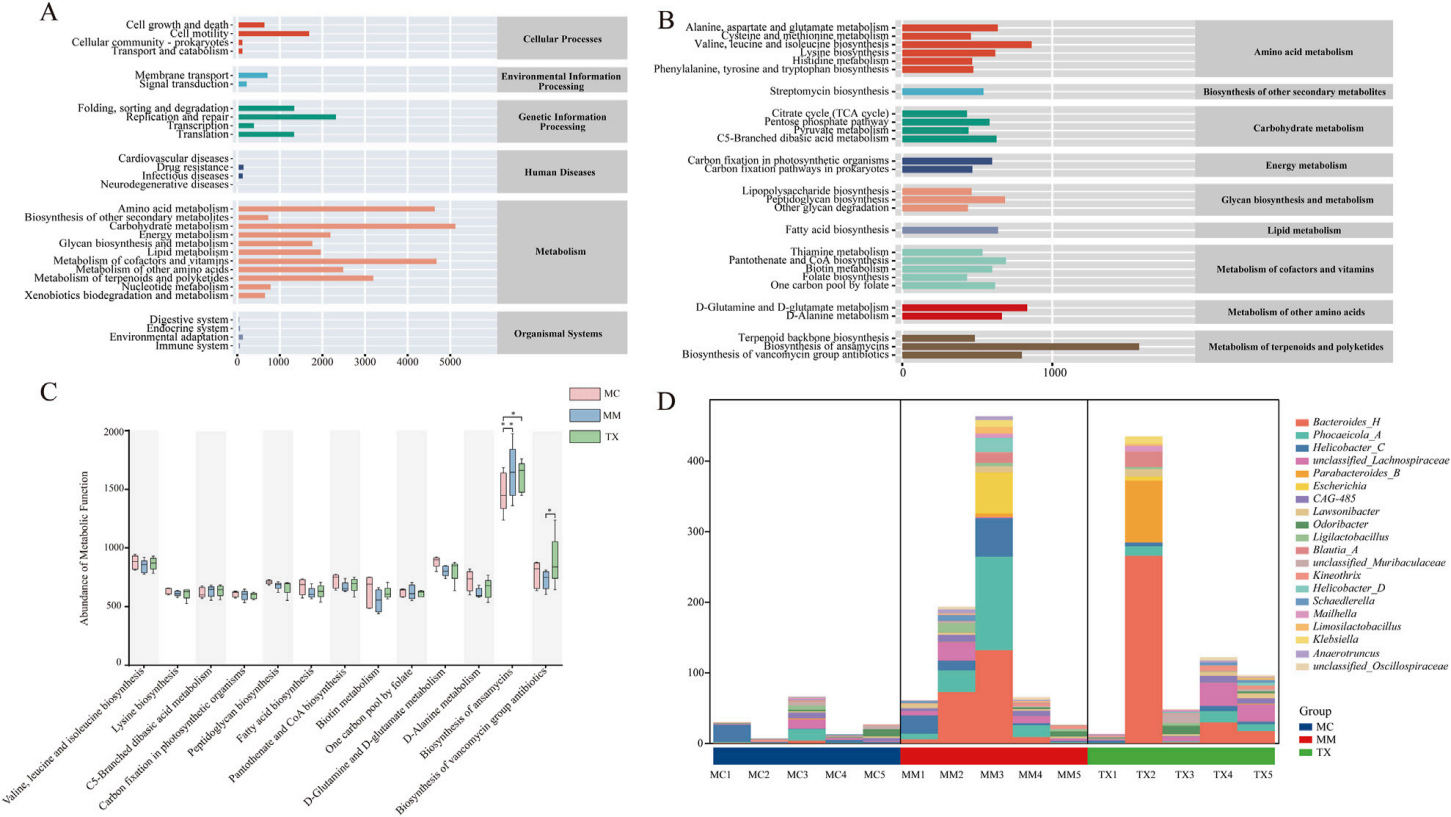
Effects of Tongxie Yaofang on the functional characteristics of colonic mucosal microbial communities in mice with diarrhea caused by liver hyperactivity with spleen deficiency pattern. **(A)** KEGG functional prediction dependency: the horizontal axis represents the abundance of KEGG functional pathways, whereas the vertical axis represents the secondary classification of these pathways. The rightmost list represents the main categories of pathways; **(B)** Metabolic pathway abundance: The horizontal axis represents the abundance of metabolic pathways, the vertical axis represents the tertiary classification of these pathways, and the rightmost list represents the secondary classification to which each metabolic pathway belongs (median >431.616); **(C)** Comparison between groups in each metabolic functional category (median >599.224). *: *p* < 0.05, **: *p* < 0.01. **(D)** Composition of bacterial communities at the genus level in the biosynthesis pathway.

Using PICRUSt2 and the KEGG database, we predicted functional and metabolic changes in the colonic mucosal microbiota of mice with liver hyperactivity with spleen deficiency pattern of diarrhea following Tongxie Yaofang treatment. At the secondary pathway level, 29 functional categories were identified (Figure 13A), encompassing cellular processes, environmental information processing, genetic information processing, human diseases, metabolism, and organismal systems. Notably, metabolic pathways presented the highest overall abundance.

Focusing on tertiary pathways with median abundances >431.6 (Figure 13B), the most prominent functions included amino acid metabolism, biosynthesis of other secondary metabolites, carbohydrate metabolism, and polysaccharide biosynthesis and metabolism. Further restriction to pathways with median abundances >599.2 (Figure 13C) revealed significant treatment effects. Modeling induced a marked increase in ansamycin biosynthesis (MC vs. MM, *p* < 0.01), which was significantly reduced by Tongxie Yaofang (*p* < 0.05). Conversely, the biosynthesis of vancomycin-containing antibiotics decreased in MM mice but returned to baseline in the TX group after treatment (*p* < 0.05).

Analysis of the microbial contributors to the altered biosynthesis pathways revealed distinct community shifts between MM and TX mice (Figure 13D). In the TX group, the pathway was predominantly associated with beneficial taxa such as *Bacteroides*, *Lachnospiraceae*, and *Parabacteroides_B*, whereas in the MM group, it was driven largely by *Phocaeicola_A* and *Helicobacter_C*. This shift suggests that Tongxie Yaofang promotes the enrichment of health-associated genera within key metabolic functions disrupted by liver hyperactivity with spleen deficiency pattern of diarrhea.

### Correlation analysis between key bacterial genera and physiological indices (MTL, CRH, TBA)

**FIGURE 14:**
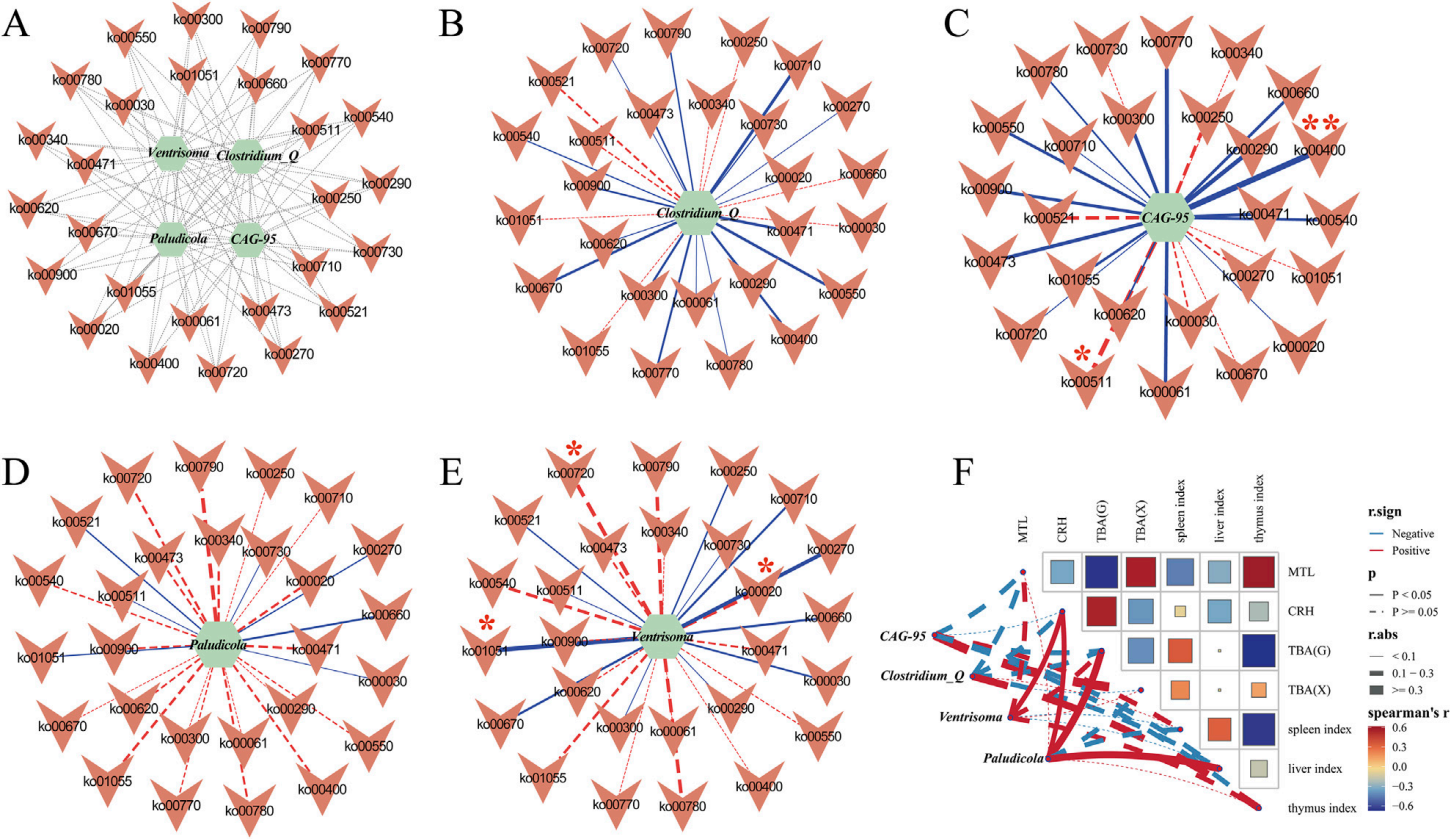
Spearman correlation analysis network diagram. **(A)** Correlation diagram between four characteristic bacteria and pathways; **(B)** Correlation analysis network diagram of *Clostridium_Q* and metabolic pathways; **(C)** Correlation analysis network diagram of *CAG-95* and metabolic pathways; **(D)** Correlation analysis network diagram of *Paludicola* and metabolic pathways; **(E)** Correlation analysis network diagram of *Ventrisoma* and metabolic pathways. *: *p* < 0.05, **: *p* < 0.01. **(F)** Correlation analysis network heatmap of the characteristic bacterial genera *Clostridium_Q, CAG-95, Paludicola, Phocea,* and *Ventrisoma* and the macroscopic indices MTL, CRH and TBA.

To investigate how characteristic genera influence host physiology and microbial metabolism, we performed Spearman correlation analysis between the top genera identified by the random forest model and various macroscopic indices (food intake, water intake, body weight, defecation frequency, and fecal moisture), as well as serum levels of MTL, CRH, and TBA. We then mapped these relationships onto a network of significantly altered KEGG pathways (Figure 14). The metabolic pathways and their numbers are shown in Supplementary Table S2.

CAG_95 was not correlated with pathway ko00790 but was strongly positively correlated with ko00400 (*p* < 0.01, ρ = 0.6991) and negatively correlated with ko00511 (*p* < 0.05, ρ = −0.5442). Ventrisoma was negatively correlated with ko00720 (*p* < 0.05, ρ = −0.5153) and ko00020 (*p* < 0.05, ρ = −0.5153) and positively correlated with ko01051 (*p* < 0.05, ρ = 0.5928). These results suggest that the pathways ko00400, ko00511, ko00710, ko00550, ko00270, ko00900, ko00670, ko00720, ko00020, and ko01051 are pivotal in modulating the colonic mucosal microbial composition and may underlie the therapeutic effects of Tongxie Yaofang (Figures 14A–E).

To delineate associations between characteristic genera and host indices—including MTL, CRH, serum TBA, liver TBA, and organ indices (spleen, liver, and thymus) — we constructed a Spearman correlation network (Figure 14F). In this network, each node represents one of the five principal genera (*Clostridium_Q*, *CAG_95*, *Paludicola* and *Ventrisoma*) or a host variable. Edges represent correlations: solid lines signify statistical significance (*p* < 0.05), dashed lines indicate nonsignificant trends, red lines indicate positive correlations, and blue lines indicate negative correlations. The node size reflects its overall connectivity and influence within the network. We found that when *p* < 0.05, *Paludicola* was positively correlated with CRH, liver TBA, and the liver index, and *Ventrisoma* was positively correlated with CRH. When *p* > 0.05, *CAG_95* showed positive, nonsignificant associations with the spleen index and serum TBA and a negative trend with the liver index. *Clostridium_Q* was primarily positively associated with the thymus index. These patterns suggest that individual genera—and their interactions — may modulate host metabolic and immune pathways integral to the therapeutic mechanism of Tongxie Yaofang in IBS-D.

## Discussions

IBS-D is a common functional gastrointestinal illness characterized by abdominal pain, irregular bowel movements, and diarrhea. Its increasing incidence and high recurrence rate impose substantial burdens on patient quality of life and healthcare costs. Aetiological factors include acute and chronic stress, brain–intestine axis dysregulation, visceral hypersensitivity, intestinal microbiota imbalance, and psychosocial disturbances; however, reliable diagnostic biomarkers and curative therapies are lacking [36].

In TCM, IBS-D falls under the broader categories of “diarrhea” and “dysentery.” Tongxie Yaofang, a classical formula from *Danxi Xinfa* (Yuan Dynasty), is indicated for liver hyperactivity with spleen deficiency pattern diarrhea and exerts multitarget, multipathway effects. However, the complexity of its constituents and their interactions has hindered the elucidation of its precise molecular mechanisms.

By integrating network pharmacology with molecular docking and molecular dynamics, we identified three primary flavonoids — kaempferol, wogonin, and nobiletin—as core bioactive molecules. All three compounds possess anti-inflammatory, antioxidant, metabolic, and immunomodulatory properties. Kaempferol inhibits NF-κB activation, suppresses the release of proinflammatory cytokines (IL-6, IL-1β, IL-18, and TNF-α), and upregulates Nrf2 expression [37–40]. Wogonin attenuates colitis by reducing TNF-α, IL-6, and IL-1β levels, stimulating ILC3-derived IL-22, and promoting mucosal barrier repair [41]. Nobiletin protects against DSS-induced colonic injury by lowering TNF-α and IL-1β levels, restoring villus architecture, and enhancing the tight junction protein claudin-7 [42]. Although these findings support the potential of individual compounds, the full therapeutic synergy of Tongxie Yaofang likely depends on the combined activity of its multiple ingredients.

Key targets — PTGS2 (COX-2), AKT1, TP53, and TNF — were enriched in the AGE-RAGE, TNF, and IL-17 signaling pathways. Molecular docking confirmed that divaricatol, naringenin, and kaempferol bind stably to PTGS2 and AKT1, suggesting that Tongxie Yaofang may inhibit COX-2 overexpression and PI3K-Akt activation. Studies have shown that COX-2 abnormalities are similar to the effects of LPS, affecting the binding of macrophages to apoptotic cells and impairing the phagocytosis of macrophages in the body. Endocytosis can prevent secondary necrosis, further inhibit inflammation, and can also reprogram macrophages to promote tissue repair [43]. Nonsteroidal anti-inflammatory drugs (NSAIDs) are a class of selective COX-2 inhibitors. NSAIDs such as mesalazine target PPARγ and NF-κB to suppress prostaglandin synthesis and TNF-α release [44]. Moreover, M1/M2 macrophage polarization in IBS involves the NF-κB, JAK/STAT, and PI3K/Akt pathways and may serve as novel therapeutic targets [45]. Thus, the multi-ingredient composition of Tongxie Yaofang likely results in broader anti-inflammatory and immunomodulatory effects than single-agent drugs.

Our *in vivo* model—*Senna alexandrina* Mill. gavage combined with restraint stress—Recapitulated key IBS-D features: reduced food and water intake; weight loss; increased defecation frequency; elevated fecal water content; histopathological damage to the liver, spleen, and colon; and altered serum CRH, motilin, and bile acid levels. Tongxie Yaofang ameliorated these signs, restored organ indices, and normalized the CRH and TBA profiles, demonstrating therapeutic efficacy.

At the genus level, *Blautia_A*, *Parabacteroides_B*, and *Bacteroides_H* predominated in the TX group. *Blautia_A* is known for its anti‐inflammatory and metabolic–modulating properties, as well as its antimicrobial activity against specific pathogens. However, reports on Blautia dynamics in intestinal disorders are conflicting: some studies have reported reduced Blautia abundance in the cecal mucosa of Crohn’s disease patients and IBS patients, whereas others have reported increased levels in certain colitis cohorts [46, 47]. To further investigate the metabolic relationship between *Blautia_A* and the bile acid profile, we found that *Blautia_A* was associated with Taurochenodeoxycholic acid (TDCA), THDCA, and Tauro-ω-muricholic acid (TωMCA) [48]. *Parabacteroides_B* exerts potent anti‐inflammatory effects *in vivo* and *in vitro* by promoting CD4^+^ T‐cell differentiation into FoxP3^+^IL‐10^+^ regulatory phenotypes and restoring mucosal barrier integrity [49]. *Parabacteroides distasonis* can ameliorate gut microbiota dysbiosis, modulate the NF-κB/MAPK and Nrf2 signaling pathways, and influence amino acid metabolism. It also regulates the expression of bile acid-related genes involved in synthesis, transport, and reabsorption in the liver and ileum, thereby alleviating alcohol-induced liver injury [50]. *Bacteroides* species are widely employed in synthetic biology applications, and the combined administration of *Bacteroides* and *Faecalibacterium* has been shown to outperform monotherapy in ameliorating experimental colitis, likely via increased phospholipid metabolism and the induction of IL‐10–producing Tregs [51]. In necrotizing enterocolitis, *Bacteroides fragilis* exhibits bile salt hydrolase (BSH) gene expression and enzymatic activity, suppresses the FXR–NLRP3 signaling pathway, restores gut microbiota dysbiosis and bile acid metabolic disorders, and thereby alleviates intestinal injury [52]. In summary, our model increased opportunistic pathogens, whereas Tongxie Yaofang treatment promoted microbial shifts that favor anti‐inflammatory, immunoregulatory, and barrier‐supportive functions.

The concept of the “intestine–liver” axis provides a modern framework for understanding the TCM “liver–spleen” relationship, with bile acids acting as key microbial–host mediators that influence neurological, endocrine, digestive, and immune functions [53]. Bile acids, which are synthesized in the liver and modified by the intestinal microbiota, regulate nutrient absorption, glucose homeostasis, and energy metabolism. From a TCM perspective, the liver governs the free flow of qi, and the gallbladder governs decision-making, reflecting their interdependence. Modern research likewise recognizes bile acids as central to lipid absorption, glycemic control, inflammation, gastrointestinal motility, maintenance of the blood–brain barrier, and neural signaling [54]. Elevated levels of chenodeoxycholic acid (CDCA) and deoxycholic acid (DCA) can induce apoptosis, disrupt tight junctions in cerebral endothelial cells, compromise the blood–brain barrier, and exacerbate neuropsychiatric symptoms [7]. Furthermore, bile acid signaling via receptors such as the farnesoid X receptor (FXR) and the G protein–coupled bile acid receptor 1 (TGR5) influences central nervous system function [55, 56].

In our model, *Senna alexandrina* Mill. gavage combined with restraint stress significantly increased the serum CRH (*p* < 0.001) and had divergent effects on bile acid pools: the serum TBA concentration decreased, whereas the hepatic TBA concentration increased in MM mice (*p* < 0.01). Tongxie Yaofang treatment reversed these changes, restoring the serum and liver TBA levels to baseline levels. These findings suggest that Tongxie Yaofang selectively normalizes disrupted bile acid metabolism in liver-depression–spleen-deficiency diarrhea, thereby modulating the intestine–liver axis.

Under physiological conditions, the intestinal microbiota orchestrates nutrient digestion and absorption in a manner analogous to the TCM concept of the spleen as the “transporter,” whereas the role of the spleen as the “guardian” reflects the key functions of the microbiota in immunity and metabolism [57]. Our network association analysis linked five characteristic genera to 29 KEGG pathways; notably, *CAG_95* showed no association with folate biosynthesis, distinguishing its metabolic profile. Correlation heatmaps revealed that *Paludicola* and *Ventrisoma* predominantly influenced CRH, hepatic bile acid levels, and the liver index. *Paludicola*, a member of the Butyricicoccaceae, remodels microbial communities by increasing short-chain fatty acid production and increasing primary bile acid levels in feces, thereby modulating bile acid signaling in the liver and ileum [58]. Recent studies indicate that altered *Phocaeicola* abundance is linked to changes in the local immune environment and colorectal pathology, while the metabolic flexibility of *P. vulgatus* highlights its importance in host–microbe interactions. In this context, *Phocaeicola* may act in a probiotic-like manner, with its increased levels under pathological conditions potentially representing a compensatory response to dysbiosis [59, 60].

Functionally, colonic mucosal microbes contributed significantly to the predicted metabolic activities (*p* < 0.05), particularly the biosynthesis of ansamycins within terpenoid and polyketide metabolism. This pathway relies on P450‐catalyzed methylene dioxygen bridge formation. Although direct links between IBS and cytochrome P450–mediated drug metabolism are lacking, P450‐targeting agents show promise in colitis models [61, 62]. Collectively, our findings indicate that Tongxie Yaofang’s efficacy in IBS-D extends beyond compositional shifts in the microbiota to the modulation of microbial secondary metabolism. We propose that its therapeutic action operates via the intestine–liver–bile acid axis, wherein bile acids suppress bile acid–sensitive bacteria and foster bile acid–dependent taxa, reshape the microbial ecosystem, activate bile acid receptors to restore intestinal immune homeostasis, and ultimately normalize liver and intestinal pathology via feedback.

This study has several limitations. First, the identification of active ingredients and disease targets relies on public databases, which are continually updated, rendering our findings time-sensitive. Second, owing to space constraints, molecular docking and dynamics simulations were limited to the top three ligand–receptor pairs and therefore may not capture all relevant interactions. Moreover, we did not experimentally validate the mRNA or protein expression of key genes within the AGE-RAGE, TNF, or IL-17 pathways. Third, our 100ns molecular dynamics simulations, while informative, may not fully represent longer-term binding dynamics. Finally, constructing the “intestine–liver–bile acid” axis solely at the levels of microbiota composition and bile acid measurements requires more precise, multiomics and metabolomic approaches to verify causal mechanisms.

## Conclusion

This work established a robust IBS-D mouse model and demonstrated the therapeutic efficacy of Tongxie Yaofang. By integrating network pharmacology, molecular docking, and molecular dynamics, we identified naringenin, divaricatol, and kaempferol as key bioactive compounds that stably bind to targets such as 5KIR and 8JOW and modulate TNF and IL-17 signaling to suppress inflammation and restore immune homeostasis. *In vivo*, IBS-D is characterized by bile acid dysregulation, impaired mucosal barrier integrity, and hepatic and splenic histopathology, all of which are ameliorated by Tongxie Yaofang. Treatment also reshaped the colonic mucosal microbiota, particularly genera such as *Clostridium_Q*, *CAG-95*, *Paludicola* and *Ventrisoma*, to normalize the serum and hepatic bile acid levels. Collectively, our findings elucidate a multitarget, microbiota-mediated mechanism by which Tongxie Yaofang alleviates IBS-D via the intestine–liver–bile acid axis, providing a scientific basis for its clinical application.

### Technical terms

Latin pharmacognostic names: *Senna alexandrina* Mill. (番泻叶 fān xiè yè); *Atractylodes macrocephala* Koidz. (白术 bái zhú); *Saposhnikovia divaricata* Schischk. (防风 fáng fēng); Paeoniae Radix Alba (白芍 bái sháo); Citri Reticulatae Pericarpium (陈皮 chén pí).

General terms: liver hyperactivity with spleen deficiency pattern(脾虚湿盛 pǐ xū shī shèng).

Book titles: Danxi Xinfa (丹溪心法 dān xī xīn fǎ).

## Data Availability

The datasets presented in this study can be found in online repositories. The names of the repository/repositories and accession number(s) can be found below: https://www.ncbi.nlm.nih.gov/, PRJNA1256748.

## Footnotes

1
https://www.91tcmsp.com/

2
https://cn.string-db.org/

3
https://www.uniprot.org/

4
https://www.genecards.org/

5
https://omim.org/

6
https://david.ncifcrf.gov/

7
http://www.bioinformatics.com.cn/

8
https://pubchem.ncbi.nlm.nih.gov/

9
https://www.rcsb.org/

10
http://greengenes.secondgenome.com/

11
https://github.com/QIIME2/q2-feature-classifier

12
https://view.qiime2.org/

13
https://www.genome.jp/kegg/
