# Supplementary material for: Deciphering the microbiological mechanism of Tongxie Yaofang in treating IBS-D: a multimodal mechanistic study in mice integrating network pharmacology, computational simulation, and 16S rRNA sequencing
Source: Exp Biol Med (Maywood). 2025 Oct 3;250:10725. doi: 10.3389/ebm.2025.10725 (PMC12531855; doi:10.3389/ebm.2025.10725)
Supplement: Supplementary file 1 [file Table1.docx]

**Table S1** Candidate compounds of Tongxie Yaofang

| No. | Herbal medicine | Chemical | OB(%) | DL |
| --- | --- | --- | --- | --- |
| 1 | Atractylodes Macrocephala Koidz. | 12-senecioyl-2E,8E,10E-atractylentriol | 62.39 | 0.22 |
| 2 | Atractylodes Macrocephala Koidz. | 14-acetyl-12-senecioyl-2E,8E,10E-atractylentriol | 60.31 | 0.3 |
| 3 | Atractylodes Macrocephala Koidz. | 14-acetyl-12-senecioyl-2E,8Z,10E-atractylentriol | 63.37 | 0.29 |
| 4 | Atractylodes Macrocephala Koidz. | α-Amyrin | 39.51 | 0.76 |
| 5 | Atractylodes Macrocephala Koidz. | (3S,8S,9S,10R,13R,14S,17R)-10,13-dimethyl-17-[(2R,5S)-5-propan-2-yloctan-2-yl]-2,3,4,7,8,9,11,12,14,15,16,17-dodecahydro-1H-cyclopenta[a]phenanthren-3-ol | 36.22 | 0.78 |
| 6 | Atractylodes Macrocephala Koidz. | 3β-acetoxyatractylone | 54.06 | 0.21 |
| 7 | Atractylodes Macrocephala Koidz. | 8β-ethoxy atractylenolide Ⅲ | 35.95 | 0.21 |
| 8 | Paeoniae Radix Alba | 11alpha,12alpha-epoxy-3beta-23-dihydroxy-30-norolean-20-en-28,12beta-olide | 64.77 | 0.37 |
| 9 | Paeoniae Radix Alba | paeoniflorgenone | 87.59 | 0.36 |
| 10 | Paeoniae Radix Alba | (3S,5R,8R,9R,10S,14S)-3,17-dihydroxy-4,4,8,10,14-pentamethyl-2,3,5,6,7,9-hexahydro-1H-cyclopenta[a]phenanthrene-15,16-dione | 43.55 | 0.53 |
| 11 | Paeoniae Radix Alba | Lactiflorin | 49.12 | 0.79 |
| 12 | Paeoniae Radix Alba | paeoniflorin | 53.87 | 0.78 |
| 13 | Paeoniae Radix Alba | paeoniflorin_qt | 68.17 | 0.39 |
| 14 | Paeoniae Radix Alba | albiflorin_qt | 66.64 | 0.32 |
| 15 | Paeoniae Radix Alba | benzoyl paeoniflorin | 31.27 | 0.74 |
| 16 | Paeoniae Radix Alba | Mairin | 55.37 | 0.77 |
| 17 | Paeoniae Radix Alba | kaempferol | 41.88 | 0.24 |
| 18 | Paeoniae Radix Alba | (+)-catechin | 54.82 | 0.24 |
| 19 | Citrus Reticulata | naringenin | 59.29 | 0.21 |
| 20 | Citrus Reticulata | 5,7-dihydroxy-2-(3-hydroxy-4-methoxyphenyl)chroman-4-one | 47.73 | 0.27 |
| 21 | Citrus Reticulata | Citromitin | 86.9 | 0.51 |
| 22 | Citrus Reticulata | nobiletin | 61.66 | 0.51 |
| 23 | Saposhnikoviae Radix | (2R,3R)-3-(4-hydroxy-3-methoxy-phenyl)-5-methoxy-2-methylol-2,3-dihydropyrano[5,6-h][1,4]benzodioxin-9-one | 68.82 | 0.66 |
| 24 | Saposhnikoviae Radix | 11-hydroxy-sec-o-beta-d-glucosylhamaudol_qt | 50.24 | 0.26 |
| 25 | Saposhnikoviae Radix | anomalin | 59.65 | 0.65 |
| 26 | Saposhnikoviae Radix | divaricatacid | 86.99 | 0.32 |
| 27 | Saposhnikoviae Radix | divaricatol | 31.65 | 0.38 |
| 28 | Saposhnikoviae Radix | Ammidin | 34.54 | 0.22 |
| 29 | Saposhnikoviae Radix | ledebouriellol | 32.05 | 0.5 |
| 30 | Saposhnikoviae Radix | phelloptorin | 43.38 | 0.27 |
| 31 | Saposhnikoviae Radix | 5-O-Methylvisamminol | 37.99 | 0.24 |
| 32 | Saposhnikoviae Radix | Phellopterin | 40.18 | 0.27 |
| 33 | Saposhnikoviae Radix Citrus Reticulata Paeoniae Radix Alba | sitosterol | 36.91 | 0.75 |
| 34 | Saposhnikoviae Radix | wogonin | 30.68 | 0.22 |
| 35 | Saposhnikoviae Radix Paeoniae Radix Alba | beta-sitosterol | 36.91 | 0.75 |
| 36 | Saposhnikoviae Radix | Mandenol | 41.99 | 0.19 |
| 37 | Saposhnikoviae Radix | isoimperatorin | 45.46 | 0.22 |
| 38 | Saposhnikoviae Radix | Prangenidin | 36.31 | 0.21 |
| 39 | Saposhnikoviae Radix | methyl icosa-11,14-dienoate | 39.66 | 0.22 |
| 40 | Saposhnikoviae Radix | Decursin | 39.26 | 0.38 |

**Table S2** Secondary metabolic pathways and their corresponding indices

| **Metabolic pathway** | **Symbol** | **Metabolic pathway** | **Symbol** |
| --- | --- | --- | --- |
| Valine, leucine and isoleucine biosynthesis | ko00250 | Cysteine and methionine metabolism | ko00270 |
| Valine, leucine and isoleucine biosynthesis | ko00290 | Lysine biosynthesis | ko00300 |
| Histidine metabolism | ko00340 | Phenylalanine, tyrosine and tryptophan biosynthesis | ko00400 |
| Streptomycin biosynthesis | ko00521 | Citrate cycle (TCA cycle) | ko00020 |
| Pentose phosphate pathway | ko00030 | Pyruvate metabolism | ko00620 |
| C5-Branched dibasic acid metabolism | ko00660 | Carbon fixation in photosynthetic organisms | ko00710 |
| Carbon fixation pathways in prokaryotes | ko00720 | Lipopolysaccharide biosynthesis | ko00540 |
| Peptidoglycan biosynthesis | ko00550 | Other glycan degradation | ko00511 |
| Fatty acid biosynthesis | ko00061 | Thiamine metabolism | ko00730 |
| Pantothenate and CoA biosynthesis | ko00770 | Biotin metabolism | ko00780 |
| Folate biosynthesis | ko00790 | One carbon pool by folate | ko00670 |
| D-Glutamine and D-glutamate metabolism | ko00471 | D-Alanine metabolism | ko00473 |
| Terpenoid backbone biosynthesis | ko00900 | Biosynthesis of ansamycins | ko01051 |
| Biosynthesis of vancomycin group antibiotics | ko01055 |  |  |
